# Supplementary material for: Ring finger protein 213 assembles into a sensor for ISGylated proteins with antimicrobial activity
Source: Nat Commun. 2021 Oct 1;12:5772. doi: 10.1038/s41467-021-26061-w (PMC8486878; doi:10.1038/s41467-021-26061-w)
Supplement: Supplementary file 1 — Supplementary information [file 41467_2021_26061_MOESM1_ESM.pdf]

Supplementary information

TITLE:

Ring Finger Protein 213 Assembles into a Sensor for ISGylated Proteins with Antimicrobial Activity

AUTHORS:

Fabien Thery[1,2,$], Lia Martina[1,2,$], Caroline Asselman[1,2,$], Yifeng Zhang[3], Madeleine Vessely[3], Heidi Repo[1,2], Koen Sedeyn[1,4], George D. Moschonas[1,4], Clara Bredow[5], Qi Wen Teo[6], Jingshu Zhang[6], Kevin Leandro[1,2], Denzel Eggermont[1,2], Delphine De Sutter[1,2], Katie Boucher[1,2,7], Tino Hochepied[8,9], Nele Festjens[1,4], Nico Callewaert[1,4], Xavier Saelens[1,4], Bart Dermaut[2,10], Klaus-Peter Knobeloch[11], Antje Beling[5,12], Sumana Sanyal[6,13], Lilliana Radoshevich[3,*], Sven Eyckerman[1,2,*], Francis Impens[1,2,7,*]

[1] VIB-UGent Center for Medical Biotechnology, VIB, Ghent, Belgium

[2] Department of Biomolecular Medicine, Ghent University, Ghent, Belgium

[3] Department of Microbiology and Immunology, University of Iowa Carver College of Medicine, Iowa City, USA

[4] Department of Biochemistry and Microbiology, Ghent University, Ghent, Belgium

[5] Charité - Universitätsmedizin Berlin, corporate member of Freie Universität Berlin and Humboldt-Universität zu Berlin, Institute of Biochemistry, Berlin, Germany

[6] HKU-Pasteur Research Pole, School of Public Health, University of Hong Kong, Hong Kong

[7] VIB Proteomics Core, VIB, Ghent, Belgium

[8] VIB Center for Inflammation Research, VIB, Ghent, Belgium

[9] Department of Biomedical Molecular Biology, Ghent University, Ghent, Belgium

[10] Center for Medical Genetics, Ghent University Hospital, Ghent, Belgium

[11] Institute of Neuropathology, Medical Faculty, University of Freiburg, Freiburg, Germany

[12] Deutsches Zentrum für Herz-Kreislauf-Forschung (DZHK), partner side Berlin, Germany

[13] Sir William Dunn School of Pathology, University of Oxford, Oxford OX1 3RE, United Kingdom

[$]These authors contributed equally.

*Correspondence: lilliana-radoshevich@uiowa.edu
sven.eyckerman@vib-ugent.be
francis.impens@vib-ugent.be

Supplementary Figure 1

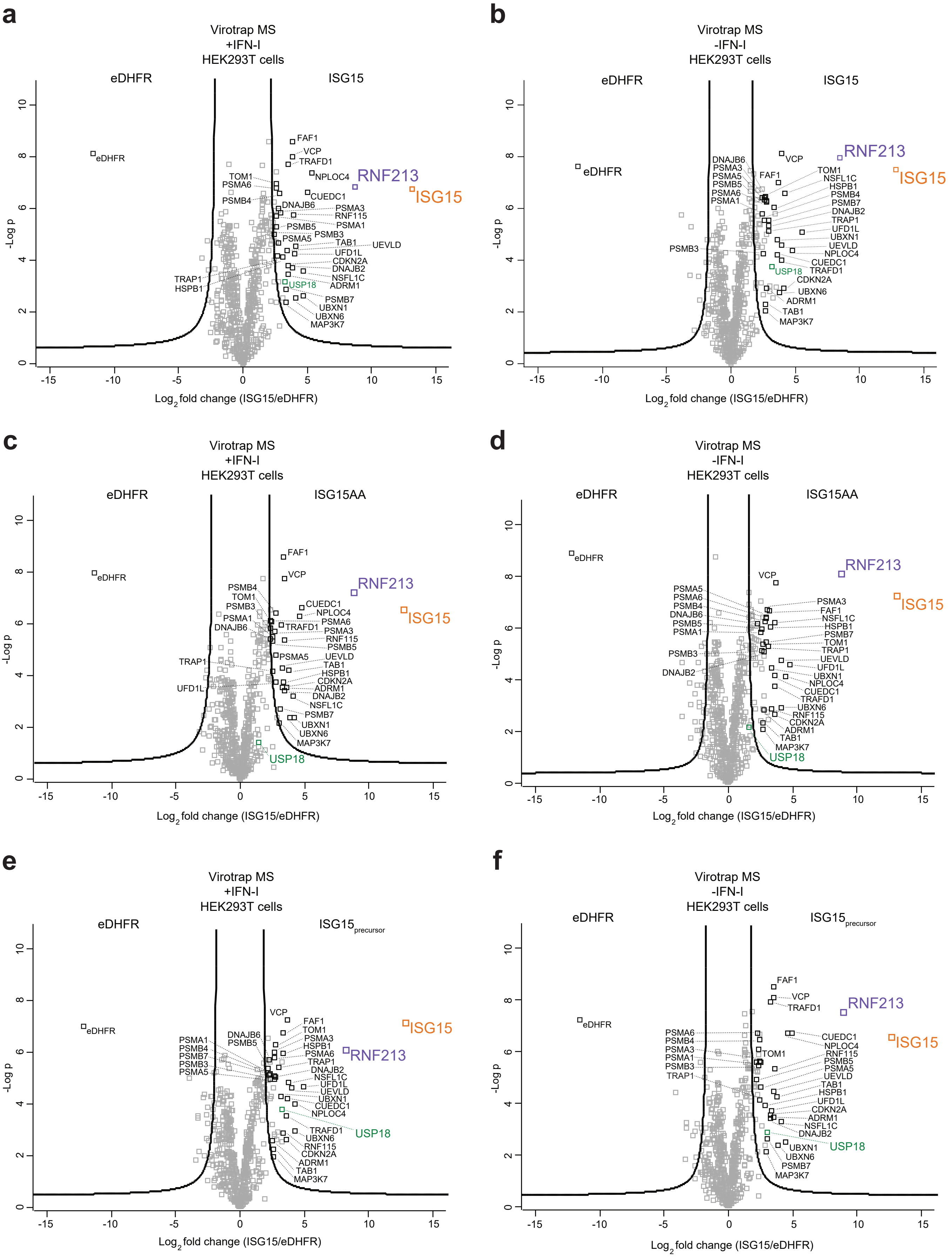

Supplementary Figure 1. RNF213 binds to premature, mature and non-conjugatable ISG15

(a-f) HIV-1 GAG protein was genetically fused to wild-type ISG15, non-conjugatable ISG15AA or the precursor form of ISG15, and transiently expressed in HEK293T cells treated or not with interferon-α (IFN-I). Virus-like particles (VLPs) were purified, lysed and protein were digested into peptides for identification and quantification by liquid chromatography tandem mass spectrometry (LC-MS/MS). Volcano plots show the result of a t-test to compare VLPs containing ISG15 versus VLPs containing *E.coli* dihydrofolate reductase (eDHFR) as negative control (n=4 replicates). The fold change (in $\log_2$) of each protein is shown on the x-axis, while the statistical significance (−log P-value) is shown on the y-axis. Proteins outside the curved lines represent specific ISG15 interaction partners. Proteins identified as common ISG15 interaction partners in all six screens are indicated (n=29) and listed in Supplementary Data 1. The volcano plot in (a) is identical to the volcano plot in Figure 1b.

Supplementary Figure 2

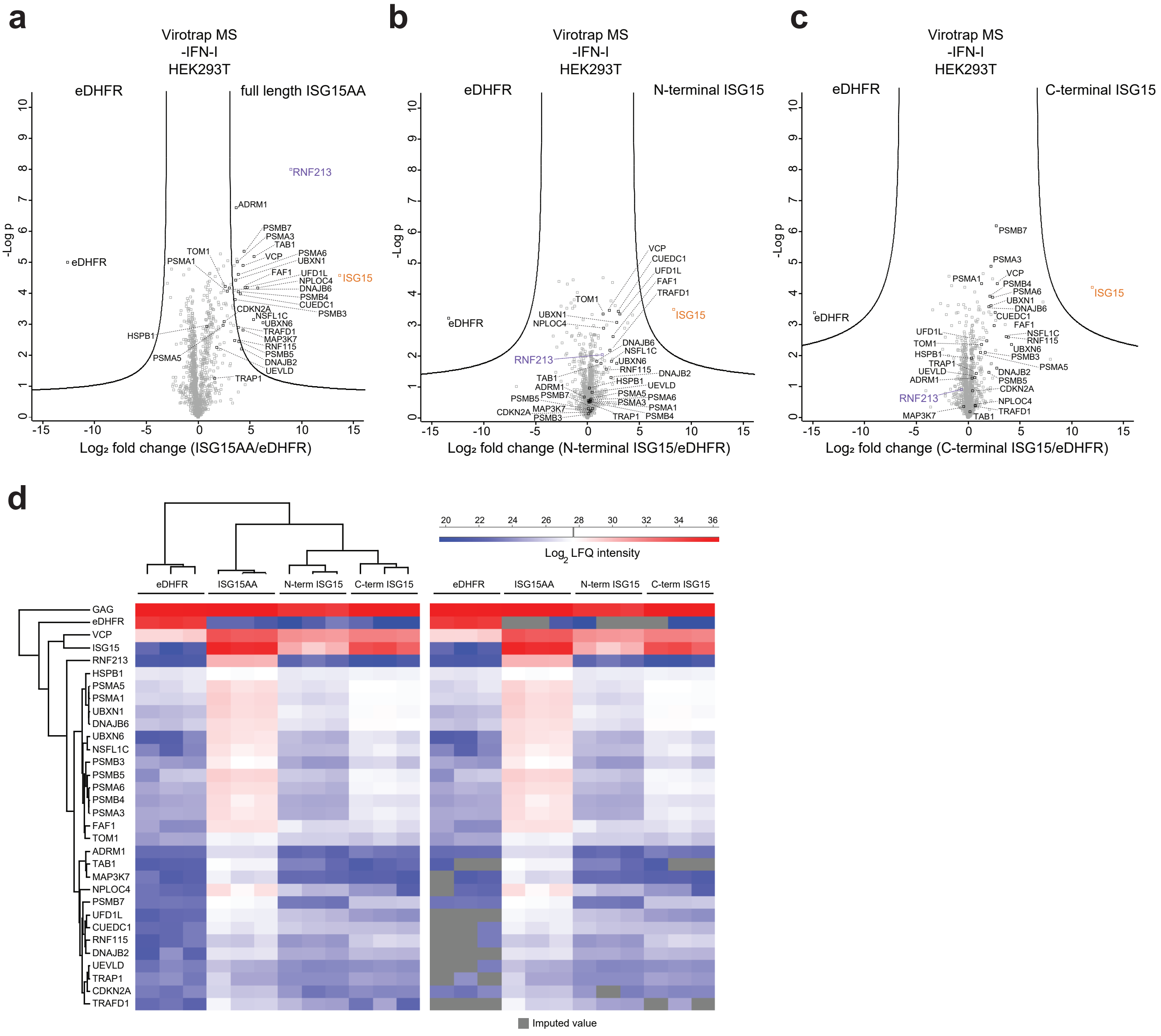

Supplementary Figure 2. Binding of RNF213 requires both N- and C-terminal domains of ISG15

(a-c) HIV-1 GAG protein was genetically fused to ISG15AA (a), the ISG15 N-terminal (b) or the ISG15 C-terminal domain (c). Constructs were transiently expressed in HEK293T and the Virotrap experiments coupled to liquid chromatography tandem mass spectrometry (LC-MS/MS) were performed as in Figure 1 and Supplementary Figure 1 (n=3 replicates). Common ISG15 interaction partners from Supplementary Figure 1 are annotated on the volcano plots and listed in Supplementary Data 1. **(d)** Heatmap showing the expression level (log_2 Label Free Quantification (LFQ) intensity) of ISG15, eDHFR, HIV-1 GAG and common ISG15 interaction partners from Supplementary Figure 1 after non-supervised hierarchical clustering. On the right side, the same heatmap is shown with originally missing values colored in gray. All common ISG15 interactors including RNF213 bind to full length ISG15, but not to the C- or N-terminal domain alone.

Supplementary Figure 3

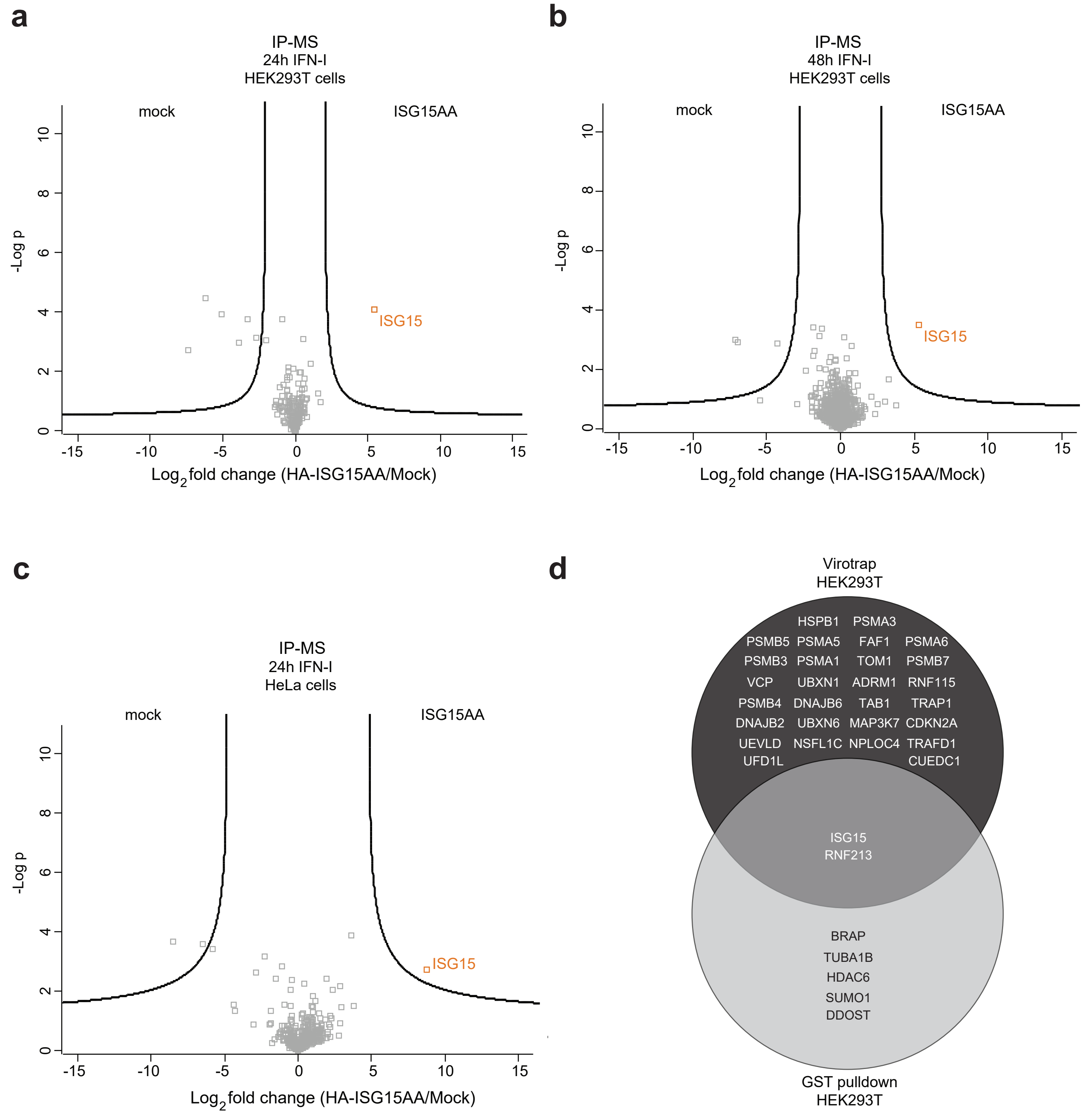

Supplementary Figure 3. ISG15 co-immunoprecipitation coupled to mass spectrometry

(a-c) Immunoprecipitation coupled to mass spectrometry (IP-MS) using HA antibodies was performed from lysates of HEK293T or HeLa cells transfected with HA-ISG15AA or mock and treated with IFN-I for 24h or 48h. Pulled down proteins were digested with trypsin on the beads prior to their identification and quantification by liquid chromatography tandem mass spectrometry (LC-MS/MS). Volcano plots show the result of a t-test to compare ISG15 pull downs versus mock control (n=3 replicates). The fold change (in $\log_2$) of each protein is shown on the x-axis, while the statistical significance (−log P-value) is shown on the y-axis. Proteins outside the curved lines represent significantly enriched proteins. Except for ISG15 itself, no interacting proteins were identified in the ISG15 pull down experiments. **(d)** Venn diagram showing the overlap between the common ISG15 interaction partners identified by Virotrap (n=29, Supplementary Figure 1) and GST (Glutathione S-Transferase) pull down in HEK293T cells (Figure 1d). Next to ISG15 itself, only RNF213 overlaps between those two type of experiments.

Supplementary Figure 4

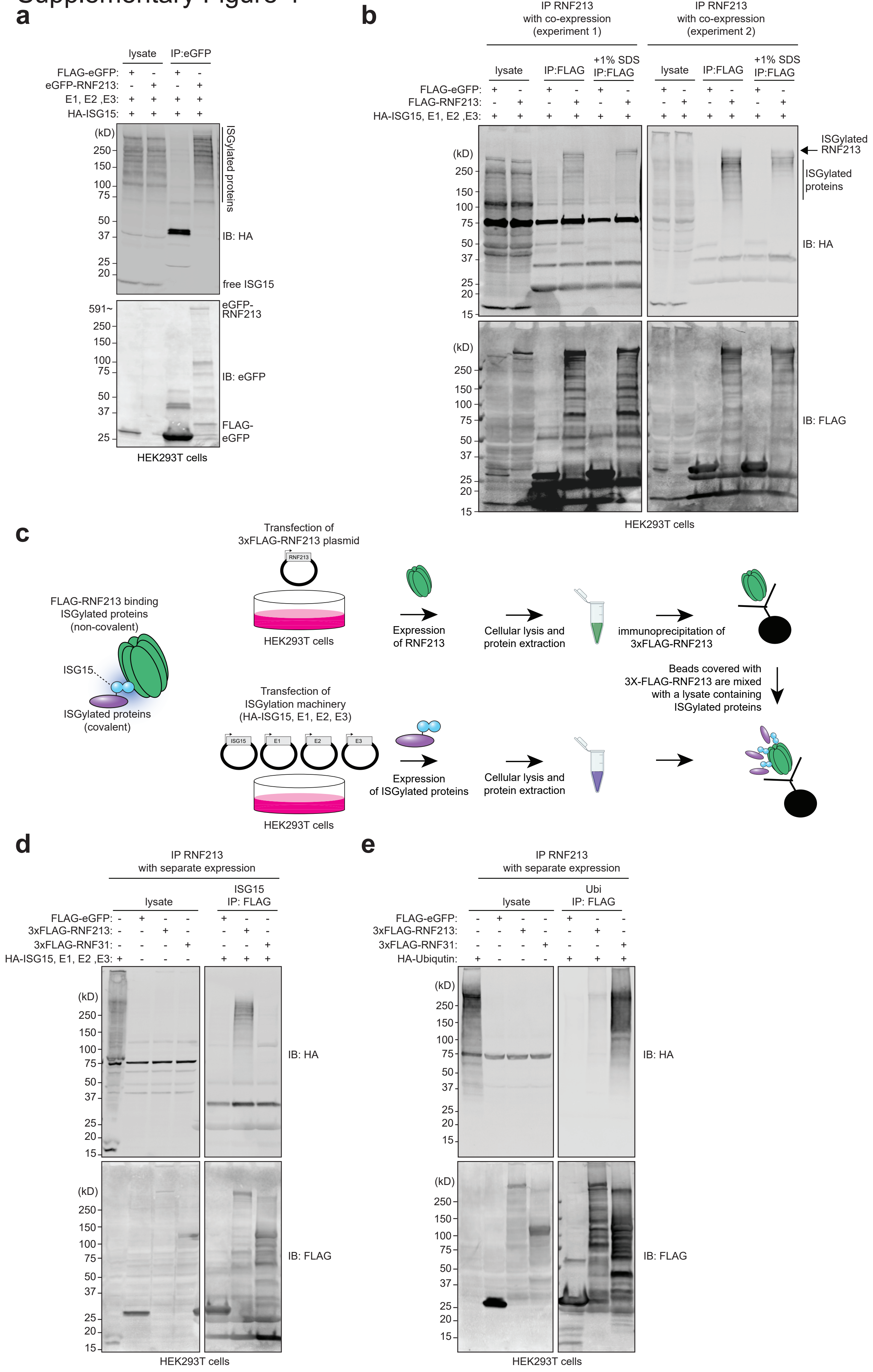

Supplementary Figure 4. RNF213 binds non-covalently to ISGylated proteins but not to ubiquitinated proteins

(a) GFP immunoprecipitation (IP) was performed from lysates of HEK293T cells expressing eGFP-RNF213 or FLAG-eGFP in combination with HA-ISG15 and the ISGylation machinery (E1, E2, E3). A smear of ISGylated co-immunoprecipitated proteins was detected by immunoblotting (IB) after IP with eGFP-RNF213, but not with FLAG-eGFP. **(b)** FLAG immunoprecipitation was performed in HEK293T cells expressing 3xFLAG-RNF213 or FLAG-eGFP in combination with HA-ISG15 and the ISGylation machinery (E1, E2, E3). After immunoprecipitation, beads were washed either with a buffer containing 1% Triton-X-100 or with a buffer containing 1% Triton-X-100 and 1 % Sodium dodecyl sulfate (SDS) to remove ISGylated proteins that were non-covalently bound to FLAG-RNF213. **(c)** Workflow showing the strategy for co-immunoprecipitation of ISGylated proteins with separate expression of FLAG-RNF213 and ISGylated proteins. FLAG immunoprecipitation is performed from a lysate of HEK293T cells expressing 3xFLAG-RNF213. After binding of FLAG-RNF213, beads are washed and subsequently mixed with a lysate of HEK293T cells expressing HA-ISG15 and the ISGylation machinery (E1, E2, E3). In this way, we can exclude that (part of) the smear of co-immunoprecipitated ISGylated proteins is actually derived from ISGylated RNF213 itself. **(d)** FLAG immunoprecipitation was performed from lysates of HEK293T cells expressing 3xFLAG-RNF213, FLAG-RNF31 (HOIP) or FLAG-eGFP as control. According to the workflow in (c), after binding of the bait protein beads were mixed with a lysate of HEK293T cells expressing HA-ISG15 and the ISGylation machinery (E1, E2, E3). While FLAG-RNF213 was capable of pulling down ISGylated proteins, FLAG-RNF31 or FLAG-eGFP were not. **(e)** FLAG immunoprecipitation of 3xFLAG-RNF213, FLAG-RNF31 or FLAG-eGFP was performed as in (d), but after binding of the bait protein beads were mixed with a lysate of HEK293T cells expressing HA-Ubiquitin. FLAG-RNF31 efficiently pulled down ubiquitinated proteins, but FLAG-RNF213 and FLAG-eGFP did not. Source data are provided as a Source Data file.

Supplementary Figure 5

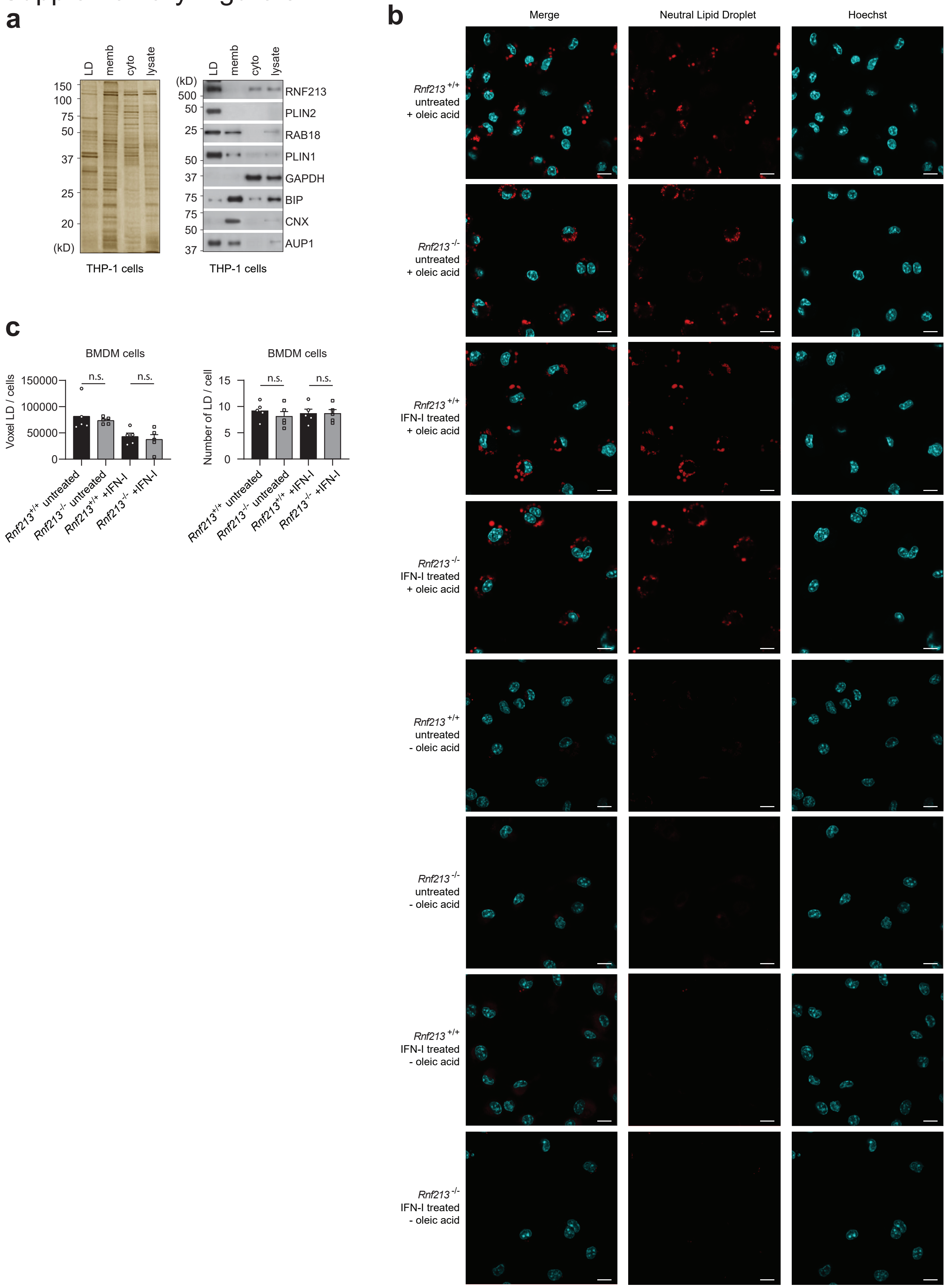

Supplementary Figure 5. RNF213 deficiency does not destabilize lipid droplets in macrophages. **(a)** THP-1 or primary human monocytes cells were cultured in the presence of 10 mM BSA-conjugated oleic acid. Lipid droplets (LDs)-enriched fractions were isolated by ultracentrifugation floatation assay on a sucrose step-gradient. Membrane (Memb) bound fraction proteins and cytosolic (Cyto) proteins were isolated by ultracentrifugation sedimentation assay. SDS-PAGE and silver staining shows equal protein loading for each fraction (left panel). Immunoblotting reveals that RNF213 is mainly associated to lipid droplet. Immunoblotting against PLIN1, PLIN2, RAB18 and AUP1 shows efficient LD isolation. Immunoblotting against CNX and BIP was used as markers for the membrane fraction. Immunoblotting against GAPDH was used as a marker for the cytosolic fraction. **(b)** Representative images of bone marrow-derived macrophages (BMDM) cells derived from *Rnf213*$^{+/+}$ or *Rnf213*$^{-/-}$ mice. Cells were treated with interferon-β, 200 μM mM BSA-conjugated oleic acid or both 24 h prior to fixation. Scale bars in the pictures are 10 microns (Hoechst = 2'-[4-ethoxyphenyl]-5-[4-methyl-1-piperazinyl]-2,5'-bi-1H-benzimidazole trihydrochloride trihydrate) . **(c)** At least 100 cells were used to count the volume (voxel, left panel) and number (right panel) of lipid droplets per cell (right panel) from five different fields containing each 20 to 30 cells. Quantification was performed with the Volocity software. No differences in volume or number of lipid droplets were observed between *Rnf213*$^{+/+}$ and *Rnf213*$^{-/-}$ cells (representative results from a single experiment, AVG ± SEM, n=5 fields, two-tailed unpaired t-test, n.s.= not significant), indicating that RNF213 deficiency does not lead to reduced stability of lipid droplets in macrophages. Source data are provided as a Source Data file.

Supplementary Figure 6

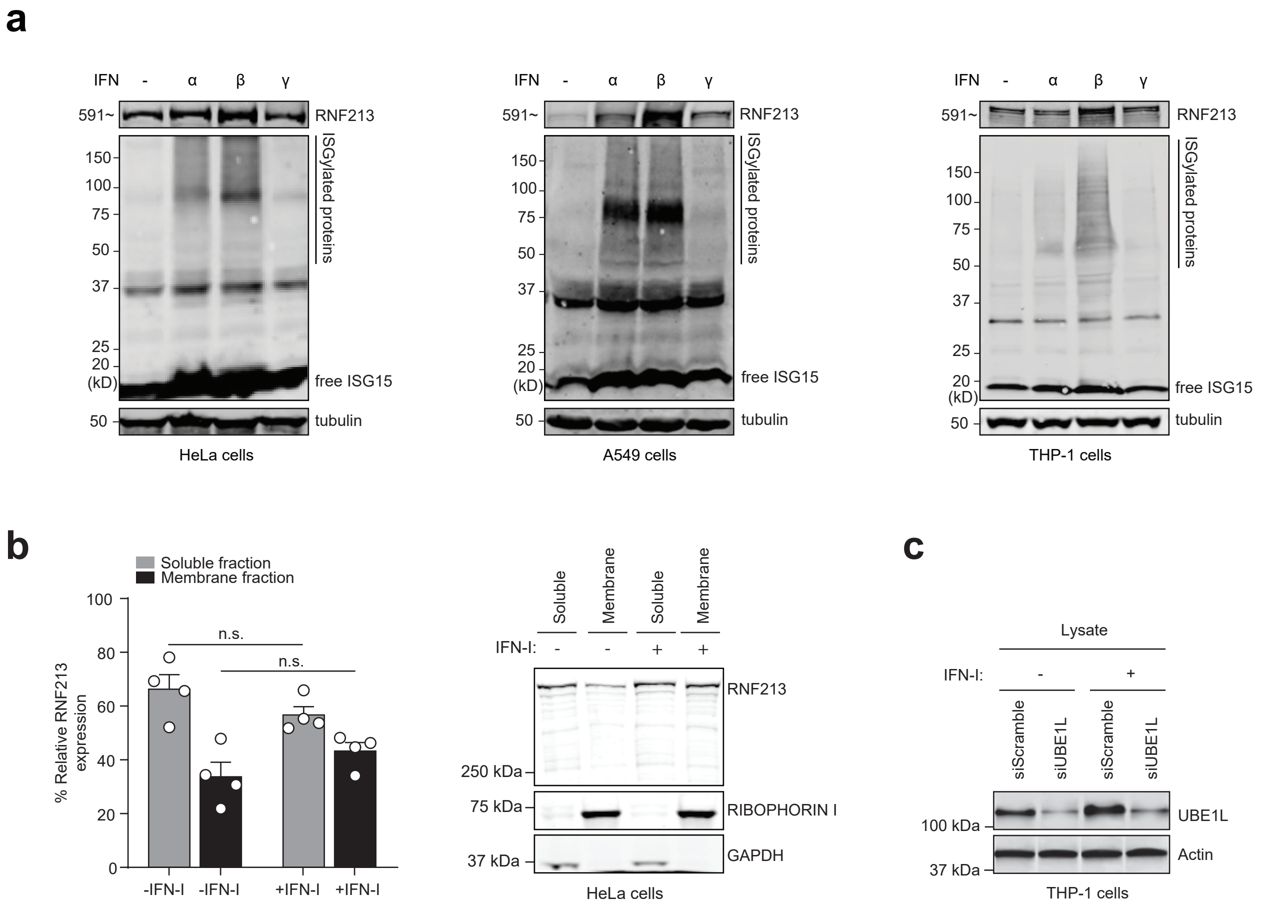

Supplementary Figure 6. IFN-I induction and regulation of RNF213 and ISG15 in human cell lines

(**a**) HeLa, A549 or THP-1 cells were either untreated or treated with the indicated type of interferon (IFN-α/β/γ) for 48 h prior to lysis and immunoblotting against RNF213, ISG15 and tubulin as loading control. RNF213 and ISG15 are co-induced, primarily by interferon-α and β. (**b**) HeLa cells were treated or not with interferon-β (IFN-I) prior to lysis and distribution of RNF213 between the soluble and membrane-associated fraction was quantified by immunoblotting. Purity of the soluble and membrane associated fractions was evaluated using GAPDH and Ribophorin I as respective marker proteins (AVG ± SEM, n=4 independent replicates, two-tailed unpaired t-test, n.s. = not significant). Interferon treatment led to a non-significant decrease and increase of RNF213 in the soluble and membrane fractions, respectively. (**c**) Immunoblots against UBE1L and tubulin as loading control confirmed knockdown of UBE1L in the experiment shown in Figure 3b. Source data are provided as a Source Data file.

Supplementary Figure 7

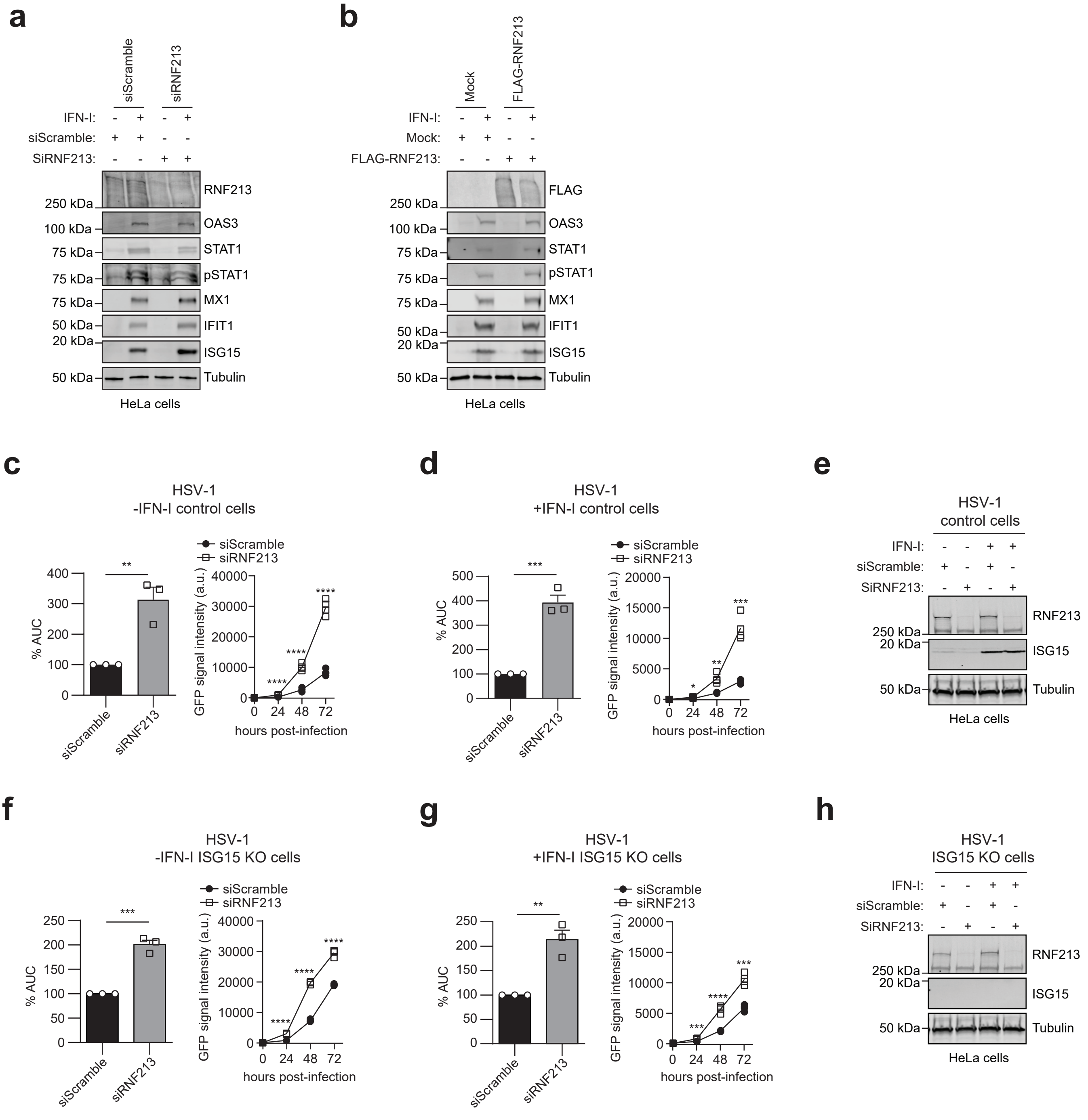

Supplementary Figure 7. RNF213 counteracts *in vitro* infection with HSV-1

(a-b) HeLa cells were transfected with a pool of siRNAs targeting RNF213 (siRNF213) or a pool of scrambled siRNAs (siScramble) as control (a). Alternatively, HeLa cells were transfected with 3X-FLAG-RNF213 or a mock plasmid as control (b). 24h hours later, cells were treated with interferon-β (IFN-I) for an additional 24 h. Immunoblotting against OAS3, STAT1, phosphorylated STAT1 (pSTAT1, Y701), MX1, IFIT1 and ISG15 confirmed that RNF213-depleted cells and RNF213-overexpressing cells efficiently induce ISG expression in response to IFN-I. Immunoblots against tubulin were used as loading control and blots against RNF213 and FLAG confirmed knockdown and overexpression, respectively. **(c-h)** Wild-type (c-e) or ISG15 knockout HeLa cells (f-h) were infected up to 72 h with eGFP-expressing recombinant herpes simplex virus 1 (HSV-1) at multiplicity of infection (MOI) 0.1. 72 h prior to infection, cells were transfected with a pool of siRNAs targeting RNF213 (siRNF213) or a pool of scrambled siRNAs (siScramble) as control. Additionally, cells were treated with interferon-α 16 h prior to infection (d, g). The viral load was determined by monitoring the GFP signal in each condition every 24 h to generate a viral growth curve (right panels in (c, d, f ,g) show a representative viral growth curve from a single experiment, n=4 technical replicates, curve connecting AVG, two-tailed unpaired t-test comparing siRNF213 to siScramble, a.u. = arbitrary units). The area under the curve (AUC) was calculated for each growth curve and the average AUC of three independent experiments is shown relative to the siScramble control (left panels in (c, d, f ,g), AVG ± SEM, n=3 independent experiments, two-tailed unpaired t-test,). Immunoblots against RNF213 and ISG15, with tubulin as loading control, confirmed knockdown of RNF213 and the absence of ISG15 in the knockout cells (e, h). Asterisks indicate p-values with * $p < 0.05$, ** $p < 0.01$, *** $p < 0.001$ and **** $p < 0.0001$. Source data are provided as a Source Data file.

Supplementary Figure 8

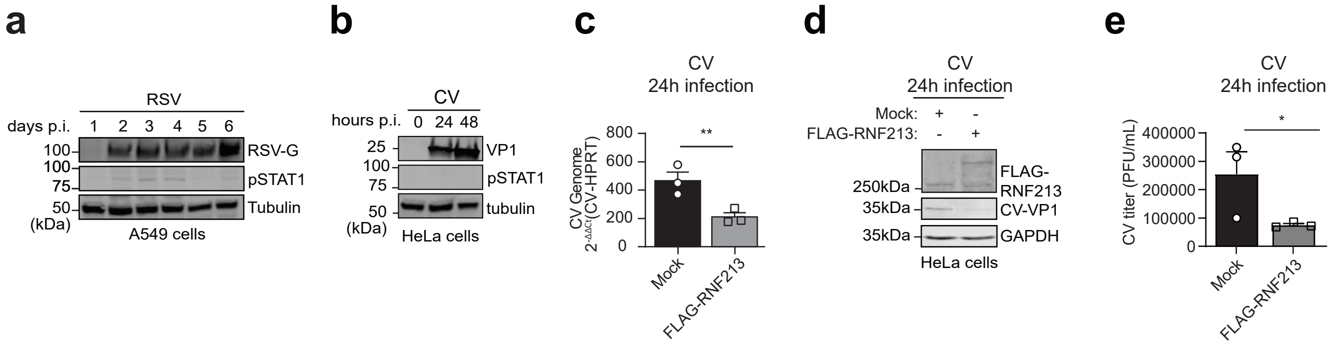

Supplementary Figure 8. RNF213 counteracts *in vitro* infection with RSV and CVB3

(a) A549 cells were infected with human respiratory syncytial virus (RSV)-A2 for up to six days at multiplicity of infection (MOI) 0.001. Immunoblotting against RSV-G confirmed infection, while pSTAT1 indicated type-I interferon signaling especially 2 to 4 days post infection. Tubulin was used as loading control. **(b)** HeLa cells were infected with coxsackievirus B3 (CV) up to 48 h at MOI 0.01. Immunoblotting against coxsackie viral protein VP1 confirmed infection, while the absence of pSTAT1 indicated no induction of type-I interferon signaling. **(c-e)** HeLa cells were infected with CV at MOI 0.01 for 24 h. 24 h prior to infection, cells were transfected with 3X-FLAG-RNF213 or a mock plasmid as control. The intracellular viral RNA load was determined by qRT-PCR (representative results from a single experiment, AVG ± SEM, n=3 technical replicates, one-tailed unpaired t-test) (c). The intracellular viral protein load was determined by immunoblotting against VP1, with GAPDH as loading control (d). Extracellular viral titers were determined by counting plaque forming units (PFUs) after serial dilution (representative results from a single experiment, AVG ± SEM, n=3 technical replicates, one-tailed unpaired t-test) (e). Overexpression of RNF213 leads to a significant decrease in CV infection as measured by lower viral genome (c), protein (d) and titer (e). Asterisks indicate p-values with * $p < 0.05$ and ** $p < 0.01$. Source data are provided as a Source Data file.

Supplementary Figure 9

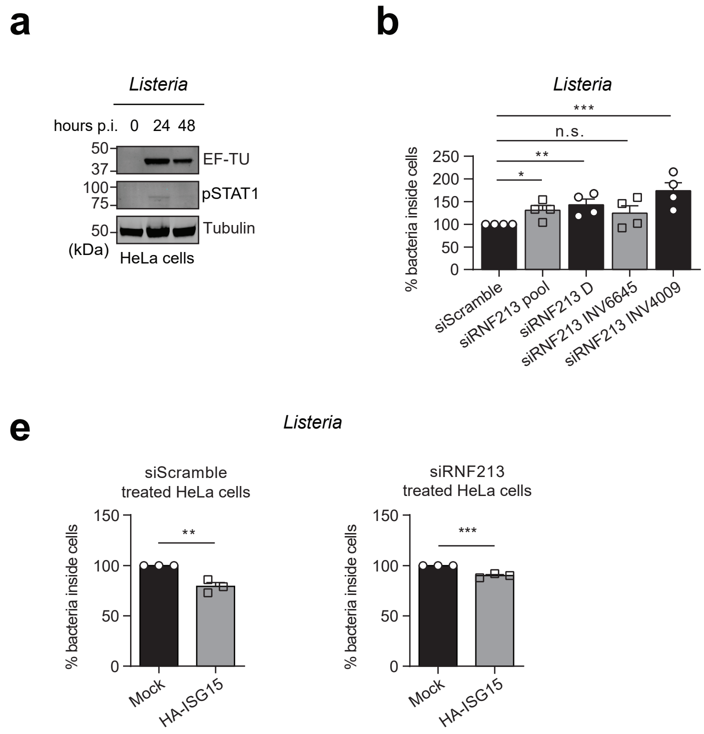

Supplementary Figure 9. RNF213 counteracts *Listeria* infection *in vitro*

(a) HeLa cells were infected with *Listeria monocytogenes* EGD (*Listeria*) and cultured for up to 48 h post infection (p.i.) at a multiplicity of infection (MOI) of 25. Immunoblotting against *Listeria* EF-TU confirmed infection, while phosphorylated STAT1 (pSTAT1, Y701) showed a slight induction of type-I interferon signaling 24 h post infection. Tubulin was used as loading control. **(b)** HeLa cells were infected with *Listeria* for 16 h at MOI 25. 24 h prior to infection, cells were transfected with a pool of siRNAs against RNF213 (siRNF213 pool, used in all other experiments) or individual siRNAs against RNF213 (siRNF213 D, siRNF213 INV6645, siRNF213 INV4009) or a pool of scrambled siRNAs (siScramble). The percentage of bacteria inside cells is shown relative to siScramble-transfected cells (AVG ± SEM, n=4 independent experiments, two-tailed unpaired t-test). **(c-d)** HeLa cells were infected with *Listeria* for 1 h at MOI 25. 24 h prior to infection, cells were transfected with plasmids encoding 3xFLAG-RNF213 or 3xFLAG-RNF213ΔC or with an empty vector (mock) as control (c). Alternatively, 24h prior to infection, cells were transfected with siRNF213 or siScramble as control (d). Intracellular *Listeria* were quantified after serial dilution by counting colony-forming units (CFUs) in a gentamycin assay (AVG ± SEM, n=4 independent experiments, two-tailed unpaired t-test). Knockdown or overexpression of RNF213 did not affect bacterial uptake. **(e)** HeLa cells were infected with *Listeria* for 16 h at MOI 25. 48 h prior to infection, cells were transfected with a pool of siRNAs targeting RNF213 (siRNF213) or a pool of non-targeting scrambled siRNAs (siScramble) as control. Additionally, 24 h prior to infection cells were transfected with a plasmid encoding HA-ISG15 or with an empty vector (mock) as control. Intracellular *Listeria* were quantified after serial dilution by counting colony-forming units (CFUs) in a gentamycin assay. The percentage of intracellular bacteria relative to mock transfected cells is shown (AVG ± SEM, n=3 independent experiments, two-tailed unpaired t-test). Asterisks indicate p-values with * $p > 0.05$, ** $p > 0.01$ and *** $p > 0.001$. Source data are provided as a Source Data file.

Supplementary Figure 10

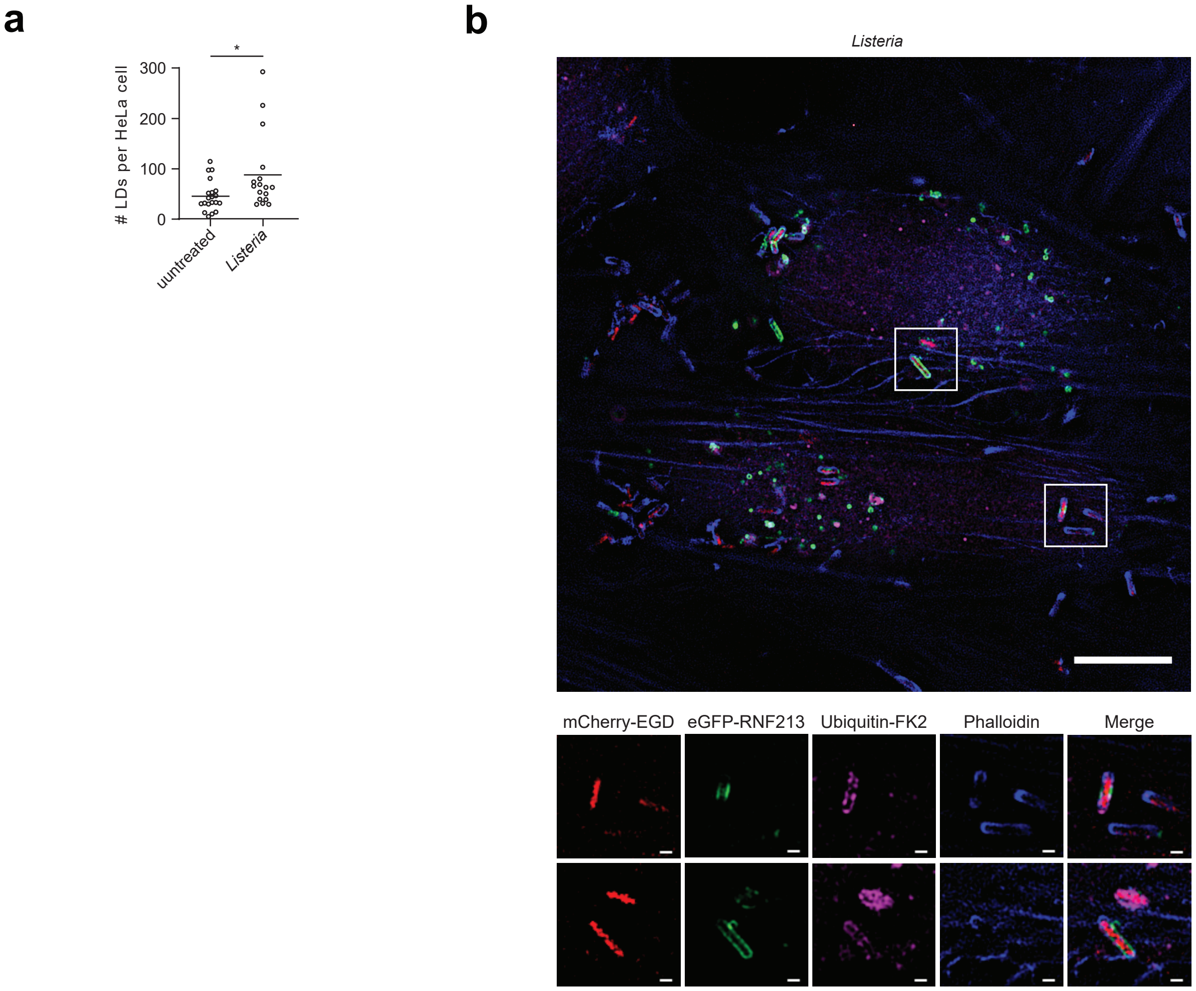

Supplementary Figure 10. RNF213 colocalizes with lipid droplets and intracellular *Listeria monocytogenes*

(a) HeLa cells transfected with eGFP-RNF213 and counterstained for lipid droplets (LDs). Following transfection cells were left untreated or infected with *Listeria monocytogenes* EGD (*Listeria*) for 24 h at a multiplicity of infection (MOI) of 25. Using microscopy images from the experiment shown in Figure 8A, lipid droplets (LDs) in uninfected (n=66 cells) and *Listeria*-infected cells (n=40 cells) were quantified with Fiji and the number of LDs per cell was calculated (representative results from a single experiment, two-tailed unpaired t-test, AVG uninfected = 46.03, AVG *Listeria*-infected = 88.24). **(b)** (upper panel) Representative image of HeLa cells infected with *Listeria monocytogenes* EGD stably expressing mCherry (*Listeria*) at a multiplicity of infection (MOI) of 20 for 6 h. Cells were transfected with eGFP-RNF213 for 48 h prior to infection, scale bar is 10 microns. (lower panels) Insets showing colocalization of ubiquitin and RNF213 to the surface of cytosolic bacteria. Actin is shown in blue, RNF213 in green, bacteria in red, and ubiquitin in magenta (FK2 antibody), scale bar is 1 micron. All images were taken with a 63× Plan Apo objective of 8-11 optical sections (0.24µm) through the middle of the cell; images were deconvolved with Zeiss Zen imaging software. Asterisk indicates the p-value with * $p < 0.05$. Source data are provided as a Source Data file.

Supplementary Figure 11

a

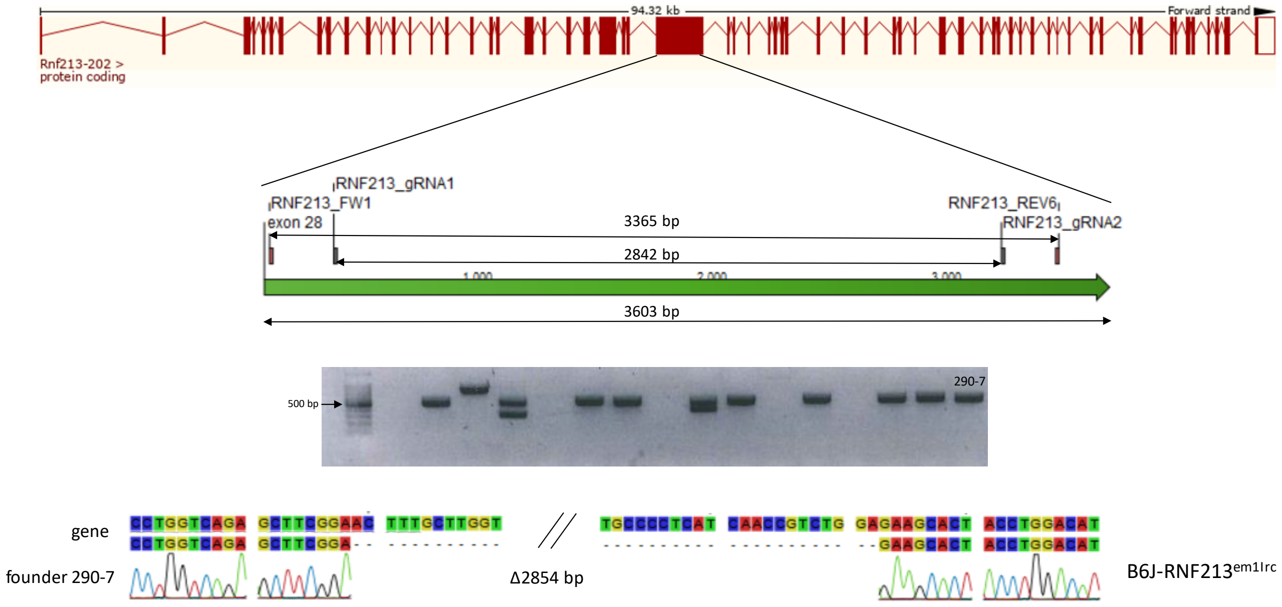

Supplementary Figure 11. CRISPR/Cas9 generation of RNF213 knockout mice

(a) Generation of C57BL/6J RNF213 KO mice. The largest exon (exon 28) of the *Rnf213* gene is 3603 bp and was targeted by 2 gRNAs, Rnf213_gRNA1 with protospacer sequence 5' CAGAGCTTCGGAACTTTGCT 3' and Rnf213_gRNA2, with protospacer sequence 5' TGTGCCCCTCATCAACCGTC 3'. The distance between the Cas9 cut sites of both gRNAs is 2842 bp. Screening for the deletion between both gRNAs was done with primers Rnf213_FW1 (5' AGTTTCTTGATCTCTTCCCC 3') and Rnf213_Rev6 (5' CTCCTCCGTCAGATCCCTA 3') generating a wild type PCR fragment of 3363 bp and a fragment of 523 bp in case of an exact deletion between both Cas9 cut sites. Several founders showed a deletion band. Founder 290-7 was further analyzed and showed a deletion of 2854 bp, resulting in a frameshift and premature stopcodons resulting in mouse line B6J-RNF213^{em1Irc}. This line was used for further breeding and the generation of *Rnf213* $^{-/-}$ and *Rnf213* $^{+/+}$ littermate control mice.

Supplementary Figure 12

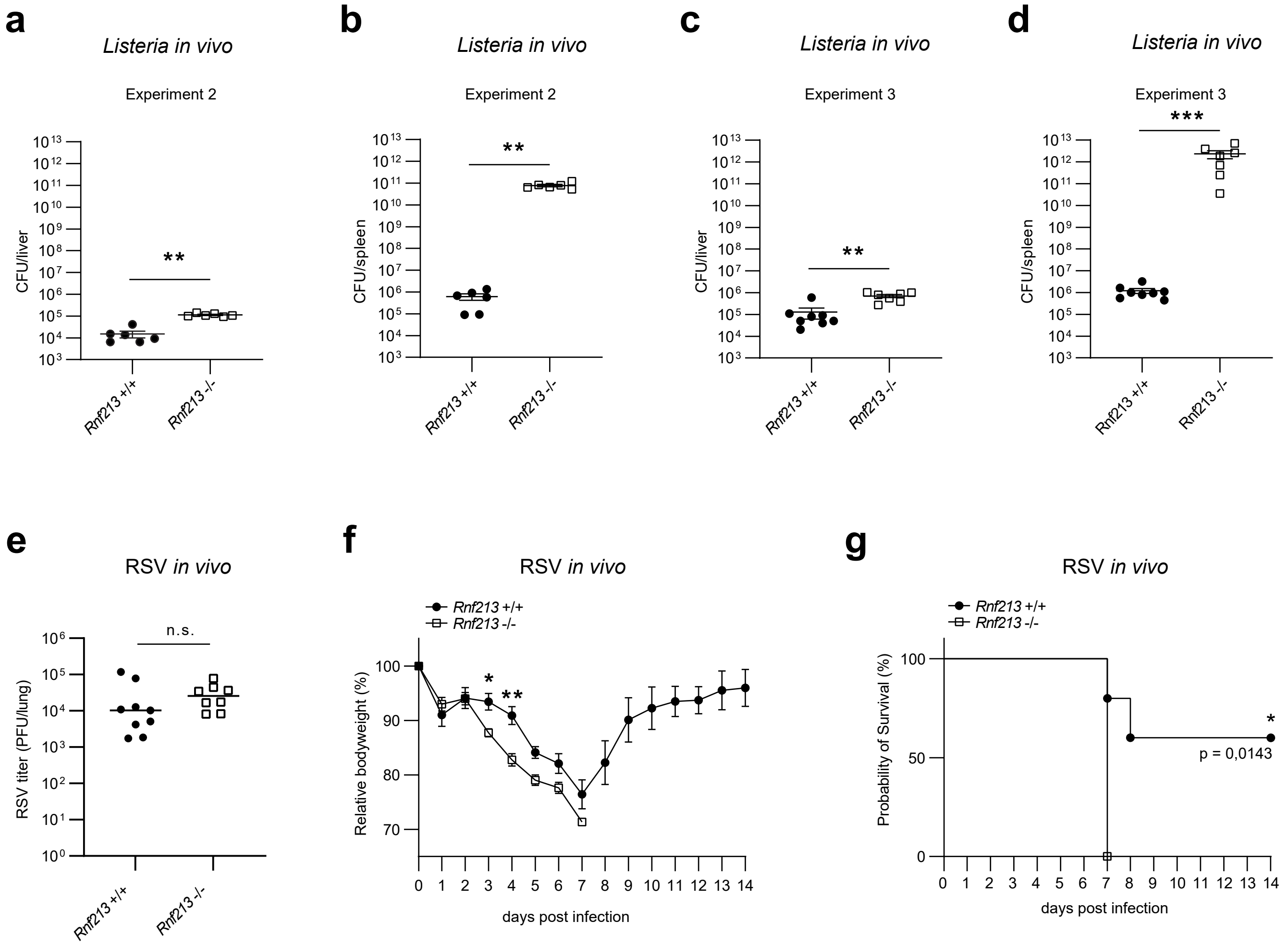

Supplementary Figure 12. RNF213 knockout mice are strongly susceptible to *Listeria* infection

(a-d) *Rnf213* $^{-/-}$ and *Rnf213* $^{+/+}$ littermates were infected intravenously with 5×10^5 *Listeria monocytogenes* EGD (*Listeria*). Liver and spleen were isolated following 72 h of infection, and CFUs per organ were counted by serial dilution and replating; dots and squares depict individual animals (representative result of a single experiment, AVG ± SEM, two-tailed Mann–Whitney test). **(a-b)** Six mice per genotypes were used in experiment 2. **(c-d)** Seven *Rnf213*$^{-/-}$ and eight *Rnf213*$^{+/+}$ mice were used in experiment 3 (note that the results of experiment 1 are shown in Fig. 9). Thus, in three independent experiments, 72 h post infection *Rnf213*$^{-/-}$ animals showed significantly higher bacterial loads in liver (~1 log) and spleen (~5 logs). **(e-g)** Twelve week old *Rnf213*$^{-/-}$ and *Rnf213*$^{+/+}$ were infected intranasally with 6 x 10^6 plaque forming units (PFUs) of in-house generated mouse-adapted human respiratory syncytial virus (RSV) after isoflurane based sedation. Seventeen mice (n=9 *Rnf213*$^{+/+}$ and n=8 *Rnf213*$^{-/-}$ littermates) were euthanized on day 5 post infection to determine the lung viral titers by serial dilution (e) (representative result of a single experiment, MED, two-tailed Mann–Whitney test, n.s. = not significant, p = 0,1742). Ten mice (n=5 for both genotypes) were used to follow-up bodyweight (f) (representative result of a single experiment, AVG ± SEM, two-way ANOVA test up-to day 7 post infection) and survival rate (g) (Mantel-Cox test) up to 14 days post infection. Asterisks indicate p-values with * $p < 0.05$, ** $p < 0.01$ and *** $p < 0.001$. Source data are provided as a Source Data file.

Supplementary Figure 13

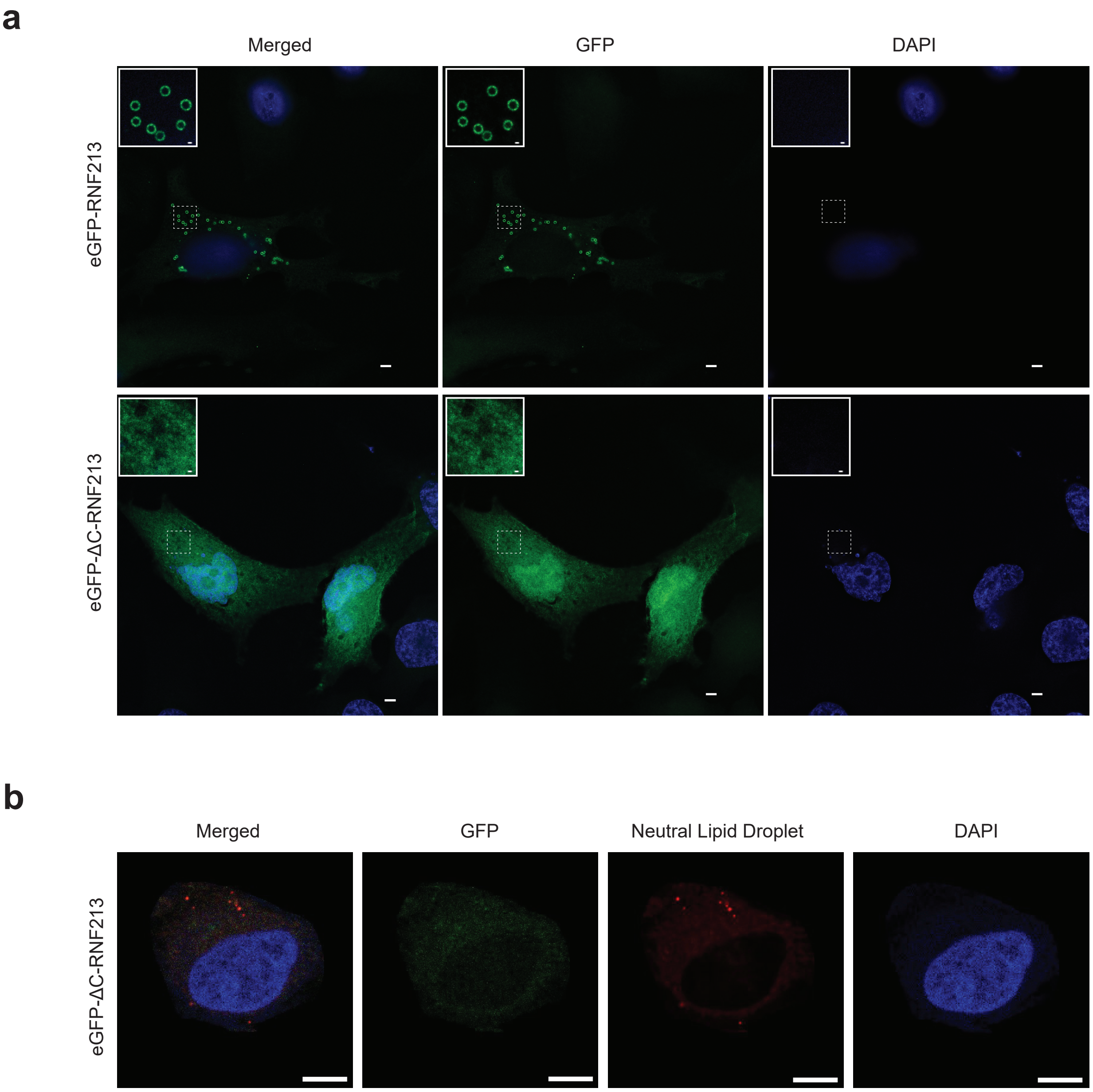

Supplementary Figure 13. The RNF213 E3 module is required for lipid droplet localization

(a) Representative images of HeLa cells transfected with eGFP-RNF213 or eGFP-RNF213ΔC for 72 hours. Following transfection cells were left untreated for 18 h and then fixed (DAPI = 4′,6-diamidino-2-phenylindole). Scale bars in the pictures and insets are respectively 10 microns and 0.5 microns. While eGFP-RNF213 adopts a spherical pattern reminiscent of lipid droplet localization reported by Sugihara et al., 2019 [1], eGFP-RNF213ΔC shows a diffuse cellular staining. **(b)** Representative images of an independent experiment in which HeLa cells were transfected with eGFP-RNF213ΔC and counterstained for lipid droplets. 72 hours post transfection cells were left untreated for 18h and then fixed. Scale bars in the pictures are 10 microns. Again, eGFP-RNF213ΔC was spread throughout the cell without co-localization to lipid droplets.

Supplementary Table 1. List of *Listeria monocytogenes* strains used in this study

Strain names	Characteristics	Collection no.	Antibiotic Resistance	Reference
EGD	*L. monocytogenes* wild-type strain	RAD0002/ BUG600		Murray *et al* [2]
EGD - mCherry	pAD-P*actA* -mCherry chromosomally integrated in EGD	RAD0058	Chloramphenicol (7.7 µg/ml)	This study***
EGDe *prfA**	*EGDe* strain expresses a constitutively active form of PrfA carrying a point mutation (G145S)	RAD0010/ BUG3057		Bécavin *et al* [3]
EGDe *prfA* ΔplcA ΔplcB Δhly*	*L. monocytogenes strain* EGDe *prfA** harboring chromosomal deletions of *plcA* , *plcB* , and *hly*	RAD0014/ BUG3648		Radoshevich *et al* [4]

Supplementary Table 2. List of plasmids used in this study.

Plasmid name	Characteristics	Collection no.	Antibiotic Resistance	Reference/accession number
pGL-T9G_MXB	mamalian expression of human MXB	/	/	Crameri *et al*[5]
pMET7-GAG-ISG15GG	Virotrap mature ISG15	/	Carbenicilline	Available upon request**
pMET7-GAG-ISG15AA	Virotrap non-conjugatable ISG15	/	Carbenicilline	Available upon request**
pMET7-GAG-ISG15precursor	Virotrap precursor ISG15	/	Carbenicilline	Available upon request**
pGL4.14-3xFLAG-RNF213	mamalian expression of human RNF213	BUG006	Carbenicilline	Available upon request*
pCMV3-FLAG-HOIP (RNF31)	mamalian expression of human RNF31	/	Carbenicilline	Addgene #50015
pRK5-HA-RNF4	mamalian expression of human RNF4	/	Carbenicilline	Addgene #59743
pMET7-Flag-eGFP	mamalian expression of eGFP	BUG001	Carbenicilline	Available upon request*
pcDNA3.1-HA-ISG15GG	mamalian expression of human mature ISG15	BUG002	Carbenicilline	Available upon request*
pcDNA3.1-HA-ISG15AA	mamalian expression of human non-conjugatable ISG15	BUG003	Carbenicilline	Available upon request*
pcDNA3-hUbe1L	Addgene #12438	/	Carbenicilline	Addgene #12438
pcDNA3.1-Ubch8	Addgene #12442	/	Carbenicilline	Addgene #12442
pTriEx2-hHERC5	Available upon request*	BUG024	Carbenicilline	Available upon request*
pDEST-eGFP-RNF213	Available upon request*	BUG016	Kanamycin	Available upon request*
pGL4.14-3xFLAG-RNF213Δ	Available upon request*	BUG043	Carbenicilline	Available upon request*
pMet7-FLAG -C-domain-RNF213	Available upon request*	BUG054	Carbenicilline	Available upon request*
pDEST-eGFP-RNF213ΔC.	Available upon request*	BUG077	Kanamycin	Available upon request*
3xFLAG-RNF213R4810K,	Available upon request*	BUG120	Carbenicilline	Available upon request*

lentiCRISPRv2 vector	Addgene #52961	/	/	Addgene #52961
psPAX2	Addgene #12260	/	/	Addgene #12260
pMD2.G	Addgene #12259	/	/	Addgene #12259
pSpCas9(BB)-2A-Puro (PX459)	Addgene #48139	/	/	Addgene #48139
pMD2.G	Addgene #12259	/	/	Addgene #12259
pcDNA3-FLAG-VSV-G	Addgene #80606	/	/	Addgene #80606
pMET7-GAG-EGFP	necessayr for GFP-positive lentiviral particles	/	Carbenicilline	Addgene #80605
pSVsport (Mock plasmid)	Thermo Fisher Scientific #10586014	BUG001	Carbenicilline	Thermo Fisher Scientific #10586014
pPL2	*L. monocytogenes* site-specific phage integration vector	BUG2176	Chloramphenicol (35 µg/ml)	Lauer *et al* [6]
pAD-P*actA* -mCherry	pPL2 expressing codon optimized mCherry under control of PactA	LRP0120	Chloramphenicol (35 µg/ml)	This study***

*Francis.impens@vib-ugent.be
**Sven.eyckerman@vib-ugent.be
***Lilliana-radoshevich@uiowa.edu

Supplementary Table 3. List of primers used for qRT-PCR, siRNA sequences used for knockdown experiment and guide RNA sequences used for the generation of knockout HeLa cell lines .

Information	Sequence/product number	Provider
**Primers**		
primer qRT-PCR for HPRT gene used in CV infection (forward)	AGTCTGGCTTATATCCAACACTTCG	Biotez (Berlin, Germany)
primer qRT-PCR for HPRT gene used in CV infection (reverse)	GACTTTGCTTTCGGTCAGG	Biotez (Berlin, Germany)
primer qRT-PCR for HPRT gene used in CV infection (probe)	TTTCACCAGCAAGCTTGCGACCTTGA	Thermo Fisher Scientific
primer qRT-PCR for CV genome used in CV infection (forward)	CCCTGAATGCGGCTAATCC	Biotez (Berlin, Germany)
primer qRT-PCR for CV genome used in CV infection (reverse)	ATTGTCACCATAAGCAGCCA	Biotez (Berlin, Germany)
primer qRT-PCR for CV genome used in CV infection (probe)	FAM-TGCAGCGGAACCG-MGB	Thermo Fisher Scientific
primer PCR for RNF213 knockout mice (forward)	AGTTTCTTGATCTCTTCCCC	Integrated DNA Technologies (ID&T) (Leuven, Belgium)
primer PCR for RNF213 knockout mice (reverse)	CTCCTCCGTCAGATCCCTA	Integrated DNA Technologies (ID&T) (Leuven, Belgium)
**siRNA**		
siRNA pool against RNF213	#M-023324-02	GE Healthcare Dharmacon
siRNA single against RNF213	#D-023324-05	GE Healthcare Dharmacon
siRNA single against RNF213	#HSS126645	Thermo Fisher Scientific
siRNA single against RNF213	#HSS184009	Thermo Fisher Scientific
siRNA non-targeting scramble	#D-001210-01-05	GE Healthcare Dharmacon
siRNA single non-targeting scramble	#D-001210-01-05	GE Healthcare Dharmacon

siRNA pool non-targeting scramble	#D-001206-13-05	GE Healthcare Dharmacon
siRNA pool against ISG15	#M-004235-04-005	GE Healthcare Dharmacon
**gRNA for Cas9 nucleases**		
gRNA1 used for ISG15 knockout HeLa cells	CACCGGAACTCATCTTTGCCAGTACAGG	Biotez (Berlin, Germany)
gRNA2 used for ISG15 knockout HeLa cells	AAACGTACTGGCAAAGATGAGTTCC	Biotez (Berlin, Germany)
gRNA1 from plasmid 1 used for RNF213 knockout HeLa cells	CACCGGAGGCAGCCTCTCTCCGCAC	Integrated DNA Technologies (ID&T) (Leuven, Belgium)
gRNA2 from plasmid 1 used for RNF213 knockout HeLa cells	AAACGTGCGGAGAGAGGCTGCCTCC	Integrated DNA Technologies (ID&T) (Leuven, Belgium)
gRNA1 from plasmid 2 used for RNF213 knockout HeLa cells	CACCGTGCAGCCCCCATAGCAGGTG	Integrated DNA Technologies (ID&T) (Leuven, Belgium)
gRNA2 from plasmid 2 used for RNF213 knockout HeLa cells	AAACCACCTGCTATGGGGGCTGCAC	Integrated DNA Technologies (ID&T) (Leuven, Belgium)
gRNA1 used for RNF213 knockout mice	CAGAGCTTCGGAACTTTGCT	Integrated DNA Technologies (ID&T) (Leuven, Belgium)
gRNA2 used for RNF213 knockout mice	TGTGCCCCTCATCAACCGTC	Integrated DNA Technologies (ID&T) (Leuven, Belgium)

Legend: guide RNA (gRNA).

Supplementary References

1. Sugihara, M., Morito, D., Ainuki, S., Hirano, Y., Ogino, K., Kitamura, A., Hirata H., Nagata, K. (2019). The AAA+ ATPase/ubiquitin ligase mysterin stabilizes cytoplasmic lipid droplets. *The Journal of cell biology*, https://doi.org/10.1083/jcb.201712120

2. Murray, E.G.D., Webb, R.A., and Swann, M.B.R. (1926). A disease of rabbits characterised by a large mononuclear leucocytosis, caused by a hitherto undescribed bacillus Bacterium monocytogenes (n. sp.). J. Pathol. Bacteriol. 29:407–439.

3. Bécavin, C., Bouchier, C., Lechat, P., Archambaud, C., Creno, S., Gouin, E., Wu, Z., Kühbacher, A., Brisse, S., Pucciarelli, M. G., García-del Portillo, F., Hain, T., Portnoy, D. A., Chakraborty, T., Lecuit, M., Pizarro-Cerdá, J., Moszer, I., Bierne, H., & Cossart, P. (2014). Comparison of widely used Listeria monocytogenes strains EGD, 10403S, and EGD-e highlights genomic variations underlying differences in pathogenicity. mBio, 5(2), e00969-14. https://doi.org/10.1128/mBio.00969-14

4. Radoshevich, L., Impens, F., Ribet, D., Quereda, J. J., Nam Tham, T., Nahori, M. A., Bierne, H., Dussurget, O., Pizarro-Cerdá, J., Knobeloch, K. P., & Cossart, P. (2015). ISG15 counteracts Listeria monocytogenes infection. eLife, 4, e06848. https://doi.org/10.7554/eLife.06848

5. Crameri, C., Bauer, M., Caduff, N., Walker, R., Steiner, F., Franzoso, F.D., Gujer, C., Boucke, K., Kucera, T., Zbinden, A., Münz, C., Fraefel, C., Greber U.F., & Pavlovic, J. (2018) MxB is an interferon-induced restriction factor of human herpesviruses. https://doi.org/10.1038/s41467-018-04379-2

6. Lauer, P., M. Y. Chow, M. J. Loessner, D. A. Portnoy, and R. Calendar. 2002. Construction, characterization, and use of two Listeria monocytogenes site-specific phage integration vectors. J. Bacteriol. https://doi.org/10.1128/JB.184.15.4177-4186.2002
