# Supplementary material for: Ring finger protein 213 assembles into a sensor for ISGylated proteins with antimicrobial activity
Source: Nat Commun. 2021 Oct 1;12:5772. doi: 10.1038/s41467-021-26061-w (PMC8486878; doi:10.1038/s41467-021-26061-w)
Supplement: Supplementary file 3 — Description of Additional Supplementary Files [file 41467_2021_26061_MOESM3_ESM.docx]

**Description of Additional Supplementary Files**

File Name: Supplementary Data 1

Description: Excel file with seven sheets listing all quantified proteins of the ISG15 Virotrap experiments shown in Fig. 1**a** and Supplementary Fig. 1**a-f**. The first row of each sheet contains a detailed description of the information listed in each column.
