# Supplementary material for: Ring finger protein 213 assembles into a sensor for ISGylated proteins with antimicrobial activity
Source: Nat Commun. 2021 Oct 1;12:5772. doi: 10.1038/s41467-021-26061-w (PMC8486878; doi:10.1038/s41467-021-26061-w)
Supplement: Supplementary file 5 — Reporting Summary [file 41467_2021_26061_MOESM5_ESM.pdf]

nature portfolio

Corresponding author(s): Lilliana Radoshevich, Sven Eyckerman, Francis Impens

Last updated by author(s): Aug 26, 2021

Reporting Summary

Nature Portfolio wishes to improve the reproducibility of the work that we publish. This form provides structure for consistency and transparency in reporting. For further information on Nature Portfolio policies, see our Editorial Policies and the Editorial Policy Checklist.

Statistics

For all statistical analyses, confirm that the following items are present in the figure legend, table legend, main text, or Methods section.

n/a	Confirmed	
☐	☒	The exact sample size (*n*) for each experimental group/condition, given as a discrete number and unit of measurement
☐	☒	A statement on whether measurements were taken from distinct samples or whether the same sample was measured repeatedly
☐	☒	The statistical test(s) used AND whether they are one- or two-sided<br>*Only common tests should be described solely by name; describe more complex techniques in the Methods section.*
☒	☐	A description of all covariates tested
☒	☐	A description of any assumptions or corrections, such as tests of normality and adjustment for multiple comparisons
☐	☒	A full description of the statistical parameters including central tendency (e.g. means) or other basic estimates (e.g. regression coefficient) AND variation (e.g. standard deviation) or associated estimates of uncertainty (e.g. confidence intervals)
☐	☒	For null hypothesis testing, the test statistic (e.g. *F*, *t*, *r*) with confidence intervals, effect sizes, degrees of freedom and *P* value noted<br>*Give P values as exact values whenever suitable.*
☒	☐	For Bayesian analysis, information on the choice of priors and Markov chain Monte Carlo settings
☒	☐	For hierarchical and complex designs, identification of the appropriate level for tests and full reporting of outcomes
☒	☐	Estimates of effect sizes (e.g. Cohen's *d*, Pearson's *r*), indicating how they were calculated

Our web collection on statistics for biologists contains articles on many of the points above.

Software and code

Policy information about availability of computer code

Data collection	Images of Listeria-infected cells were acquired using an inverted wide-field fluorescence microscope (Axio Observer 7, Carl Zeiss Microscopy) equipped with an Axiocam 506 mono camera and ZEN 2.3 Pro software. Images of lipid droplets in BMDMs were acquired using a confocal laser scanning microscope (LSM880 with Airyscan, Carl Zeiss Microscopy) using ZEN 2.3 Pro software.
Data analysis	Images of lipid droplets in BMDMs and where indicated images of infected cells were deconvolved using Zen 3.1 (Blue Edition). The number and relative area of lipid droplets in BMDMs were analyzed using Volocity 6.0. Lipid droplets in infected cells were quantified using Fiji ImageJ 1.52e. RSV plaque sizes were quantified using Fiji ImageJ 1.51n. Proteomics data analysis was performed with MaxQuant 1.6.3.4 and Perseus 1.6.2.3. All data were tested for statistical significance with GraphPad Prism 9.2.0.

For manuscripts utilizing custom algorithms or software that are central to the research but not yet described in published literature, software must be made available to editors and reviewers. We strongly encourage code deposition in a community repository (e.g. GitHub). See the Nature Portfolio guidelines for submitting code & software for further information.

Data

Policy information about availability of data

All manuscripts must include a data availability statement. This statement should provide the following information, where applicable:

- Accession codes, unique identifiers, or web links for publicly available datasets
- A description of any restrictions on data availability
- For clinical datasets or third party data, please ensure that the statement adheres to our policy

The proteomics data has been deposited in the PRIDE database, accession codes are in the data availability statement. The source data underlying all immunoblots and graphs are available in the Source Data file. Materials can be obtained from the corresponding authors upon request.

Field-specific reporting

Please select the one below that is the best fit for your research. If you are not sure, read the appropriate sections before making your selection.

- [x] Life sciences
- [ ] Behavioural & social sciences
- [ ] Ecological, evolutionary & environmental sciences

For a reference copy of the document with all sections, see nature.com/documents/nr-reporting-summary-flat.pdf

Life sciences study design

All studies must disclose on these points even when the disclosure is negative.

Sample size	No statistical methods were used to predetermine sample size for experimentation. Given the minimal variation in clonal cell lines and (littermate) lab mice, proteomics experiments included 3 (GST pull downs) or 4 (Virotrap) replicates/condition and animal experiments contained at least 5 animals/group. For all other experiments a minimum of three technical replicates were used per sample.
Data exclusions	No data points were excluded.
Replication	Experiments were repeated at least three times to be sure that the data shown in the paper is reproducible. Either the result of multiple experiments was used to calculate average values, or representative experiments are shown, as indicated.
Randomization	Cells and animals were randomly allocated to experimental groups, except when different genotypes (i.e. knockouts) were used, as indicated.
Blinding	Imaging data were quantified using Volocity, Fiji and ImageJ software, hence manual counting of samples by a blinded operator was avoided.

Reporting for specific materials, systems and methods

We require information from authors about some types of materials, experimental systems and methods used in many studies. Here, indicate whether each material, system or method listed is relevant to your study. If you are not sure if a list item applies to your research, read the appropriate section before selecting a response.

Materials & experimental systems

n/a	Involved in the study	
☐	☒	Antibodies
☐	☒	Eukaryotic cell lines
☒	☐	Palaeontology and archaeology
☐	☒	Animals and other organisms
☒	☐	Human research participants
☒	☐	Clinical data
☒	☐	Dual use research of concern

Methods

n/a	Involved in the study	
☒	☐	ChIP-seq
☒	☐	Flow cytometry
☒	☐	MRI-based neuroimaging

Antibodies

Antibodies used

The following primary antibodies were used for immunoblotting: mouse monoclonal anti-ISG15 (1:1,000; F-9, sc-166755, Santa Cruz Biotechnology), rabbit monoclonal anti-ISG15 (1:1,000; Abcam [EPR3446] ab133346;), mouse monoclonal anti-ubiquitin (1:1,000; P4D1, #sc-8017, Santa Cruz Biotechnology), mouse monoclonal anti-α-tubulin (1:1,000; B-7, #sc-5286, Santa Cruz Biotechnology), mouse monoclonal anti-GST (1:1,000; B-14, #sc-138, Santa Cruz Biotechnology), rabbit polyclonal anti-GAPDH (1:1,000; FL-335, #sc-25778, Santa Cruz Biotechnology), rabbit polyclonal anti-HA-tag (1:1,000; #H6908, Merck), rabbit polyclonal anti-RNF213 (1:1,000; #HPA003347, Merck), mouse monoclonal anti-RNF213 (1:1,000; clone 5C12; Santa Cruz Biotechnology), mouse monoclonal anti-FLAG-tag (1:5,000; M2, #F3165, Merck), rabbit polyclonal anti-LC3B (1:1,000; #PA146286, Thermo Fisher Scientific), rabbit polyclonal anti-tubulin-α (1:1,000; #ab18251, Abcam), anti-p24 GAG (1:1,000; #ab9071, Abcam), rabbit polyclonal anti-PLIN1 (1:1,000; #ab3526, Abcam), rabbit anti-PLIN2 (1:1,000; #ab108323, Abcam), rabbit polyclonal anti-Rab18 (1:1,000; #ab119900, Abcam), rabbit polyclonal anti-AUP1 (1:1000; #ab224242, Abcam), rabbit polyclonal anti-BiP (1:1,000; Abcam ab21685), rabbit polyclonal anti-Calnexin (1:1,000; #ab10286, Abcam), rabbit polyclonal anti-ATGL (1:1,000; Cell Signaling Technology #2138), goat polyclonal antibody anti-Ribophorin I (1:1,000; #sc-12164, Santa Cruz Biotechnology), mouse monoclonal anti-STAT1 (1:1,000; C-136, #sc-464, Santa Cruz Biotechnology), rabbit monoclonal anti-IFIT1 (1:1,000; #14769, Cell Signaling Technology), rabbit monoclonal phospho-STAT1 (Tyr701) (1:1,000; 58D6, #9167, Cell Signaling Technology), rabbit polyclonal anti-MxA (1:5,000; #H00004599-D01P, Novus Biologicals), rabbit polyclonal anti-MXB (1:1,000; #HPA030235, human protein atlas) and rabbit polyclonal anti-OAS3 (1:1,000; #41440, Cell Signaling Technology). Listeria EF-Tu was immunoblotted with rabbit polyclonal antisera raised against α-EF-Tu 99. Herpes Simplex virus-1 VP5 was immunoblotted with rabbit polyclonal anti-NC-1 antiserum specific for HSV-1 VP5 100. Respiratory syncytial virus RSV-G was immunoblotted with commercially available goat polyclonal anti-RSV serum (1:1,1000; #AB1128, Merck). Coxsackievirus VP1 was immunoblotted with commercially available mouse monoclonal anti-VP1 (1:1,000; 3A8, Mediagnost). Aforementioned primary antibodies were revealed using goat polyclonal anti-mouse-IgG (1:5,000; IRDye® 800CW, Li-COR), goat polyclonal anti-rabbit-IgG (1:5,000; IRDye® 800CW, Li-COR), goat polyclonal anti-mouse-IgG (1:5,000; IRDye® 680RD, Li-COR) or goat polyclonal anti-rabbit-IgG (1:5,000; IRDye® 680RD, Li-COR), except for anti-RSV serum which was revealed with secondary anti-goat

	(1:1,000; #sc-2020, Santa Cruz biotechnology). For microscopy, cells were stained with an anti-ubiquitin FK2 antibody (1:100; #ST1200, Merck) with Goat anti-mouse IgG (H+L) Superclonal™ Recombinant Secondary Antibody, Alexa Fluor 647 (1:1,000; #A28181, Thermo Fisher Scientific). To visualize intracellular Listeria, cells were stained with anti-R11 (1:200; gift from the Cossart laboratory, raised in house) and Goat anti-rabbit IgG (H+L) Superclonal™ Recombinant Secondary Antibody, Alexa Fluor 647 (1:1,000; #A27040, Thermo Fisher Scientific).
Validation	All commercial antibodies were used according to manufacturers' instructions without further validation, except for anti-RNF213 and anti-ISG15 antibodies which were verified by knockout (Figure 7). The R11 antibody is validated through its use in many studies by the Cossart lab and others and the original study in which the antibody was raised is cited in the methods.

Eukaryotic cell lines

Policy information about cell lines

Cell line source(s)	Hek293T cells originate from (Lin et al., 2014) while HeLa cells (ATCC® CCL-2™), A549 cells (ATCC® CCL-185™) THP-1 cells (ATCC® TIB-202™) and primary human CD14+ monocytes (ATCC® PCS-800-010™) were purchased from ATCC.
Authentication	Hek293T, HeLa, A549 and THP-1 cells were authenticated by PCR single locus technology by Eurofins on March 20, 2019.
Mycoplasma contamination	We consistently and regularly test for Mycoplasma using kits and PCR which is even more sensitive. All of our lines are negative.
Commonly misidentified lines (See ICLAC register)	We used only cell lines that were derived from ATCC, or in the case of 293T HEK the cells were derived from the neighbouring Callewaert lab who also obtained the cells from ATCC (see Lin et al., Nature Communications 2014). None of these cell lines are listed as commonly misidentified on ICLAC.

Animals and other organisms

Policy information about studies involving animals; ARRIVE guidelines recommended for reporting animal research

Laboratory animals	We used C57BL/6J mice that were wild type or knockout for RNF213. Both male and female mice were used in all experiments. For RSV infections twelve-week old mice were used, and for Listeria infections mice between 8 and 12 weeks of age were used, as specified in the methods and figure legends (Figure 9 and Supplementary Figure 12).
Wild animals	The study did not involve wild animals.
Field-collected samples	The study did not involve samples collected from the field.
Ethics oversight	Animal experiments were carried out in an animal facility that operates under the Flemish Government License Number LA1400536. All experiments were done under conditions specified by law and authorized by the UGent Institutional Ethical Committee on Experimental Animals.

Note that full information on the approval of the study protocol must also be provided in the manuscript.
