# Supplementary material for: Ring finger protein 213 assembles into a sensor for ISGylated proteins with antimicrobial activity
Source: Nat Commun. 2021 Oct 1;12:5772. doi: 10.1038/s41467-021-26061-w (PMC8486878; doi:10.1038/s41467-021-26061-w)
Supplement: Supplementary file 6 — Source Data [file 41467_2021_26061_MOESM6_ESM.zip › Source data_2021.08.26/Blots/Figure 2/Figure 2B.pdf]

Figure 2

Panel B

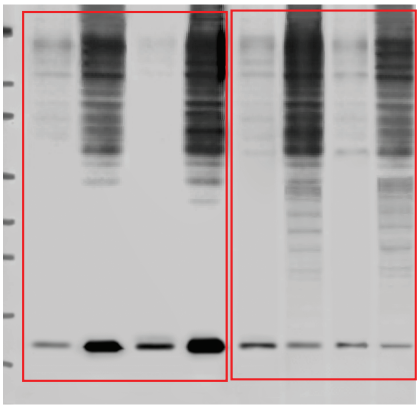

IB: ISG15

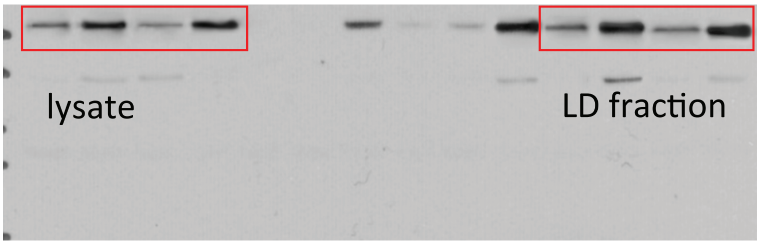

IB: RNF213

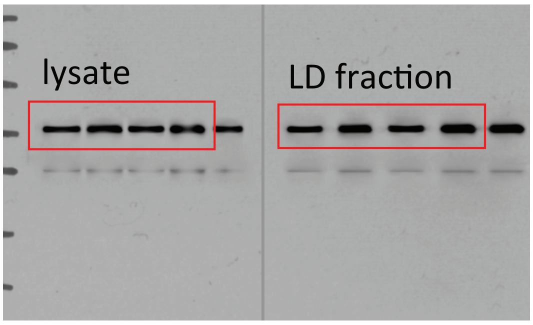

IB: Perilipin 1

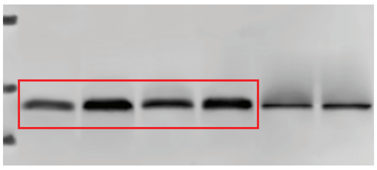

IB: Perilipin 2 (left panel)

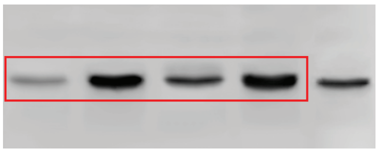

IB: Perilipin 2 (right panel)

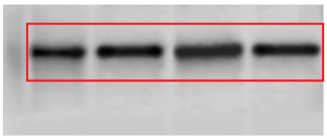

IB: GAPDH

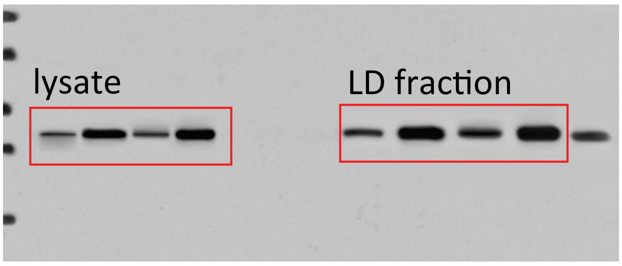

IB: ATGL
