# Supplementary figures and images for: Ring finger protein 213 assembles into a sensor for ISGylated proteins with antimicrobial activity
Source: Nat Commun. 2021 Oct 1;12:5772. doi: 10.1038/s41467-021-26061-w (PMC8486878; doi:10.1038/s41467-021-26061-w)

# Figure 10

## Panel A

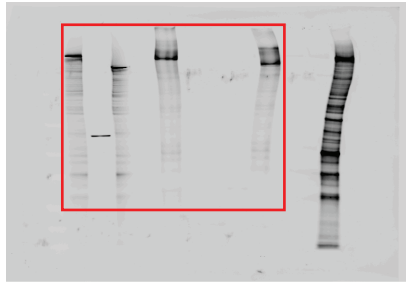

IB: FLAG

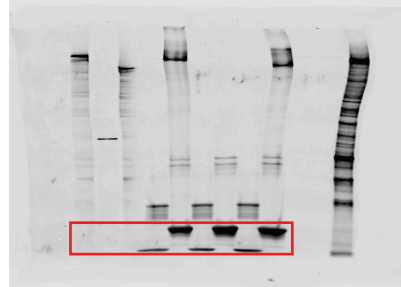

IB: GST

Supplement: Supplementary file 6 — Source Data [file 41467_2021_26061_MOESM6_ESM.zip › Source data_2021.08.26/Blots/Figure 10/Figure 10A.pdf]

# Figure 10

## Panel B

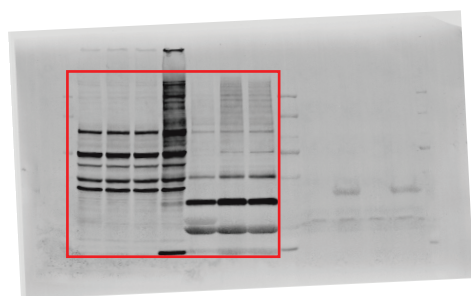

IB: HA

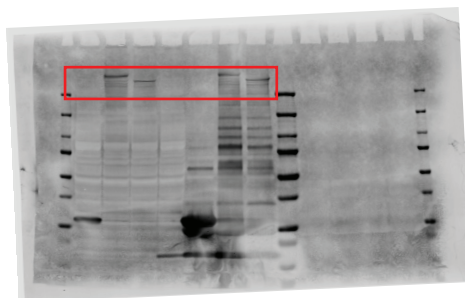

IB: FLAG

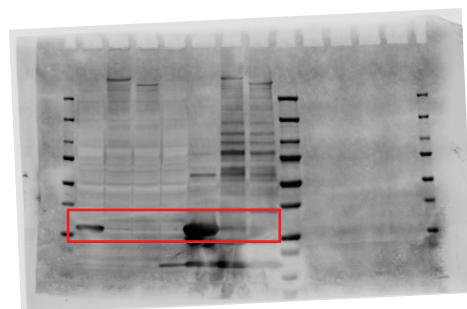

IB: FLAG

Supplement: Supplementary file 6 — Source Data [file 41467_2021_26061_MOESM6_ESM.zip › Source data_2021.08.26/Blots/Figure 10/Figure 10B.pdf]

# Figure 10

## Panel C

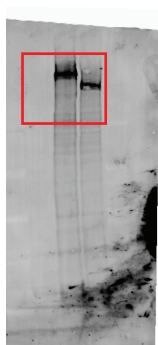

IB: FLAG

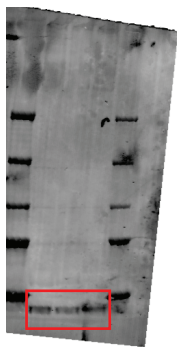

IB: Tubulin

Supplement: Supplementary file 6 — Source Data [file 41467_2021_26061_MOESM6_ESM.zip › Source data_2021.08.26/Blots/Figure 10/Figure 10C.pdf]

# Figure 1

## Panel C

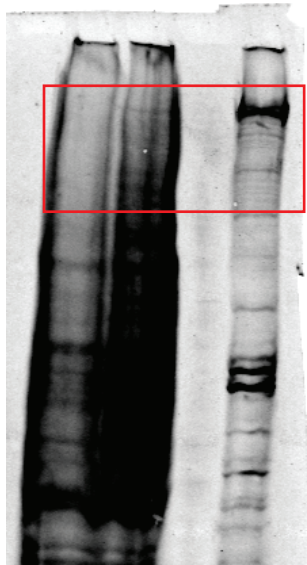

IB: RNF213

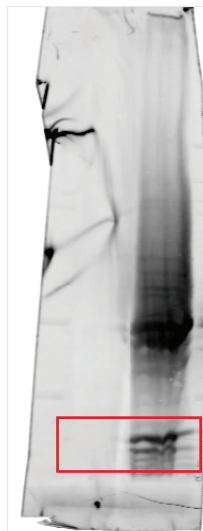

IB: ISG15

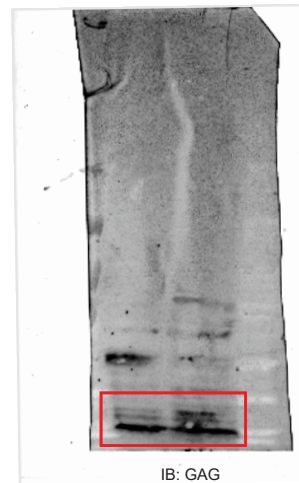

IB: GAG

Supplement: Supplementary file 6 — Source Data [file 41467_2021_26061_MOESM6_ESM.zip › Source data_2021.08.26/Blots/Figure 1/Figure 1C.pdf]

# Figure 1

Panel G

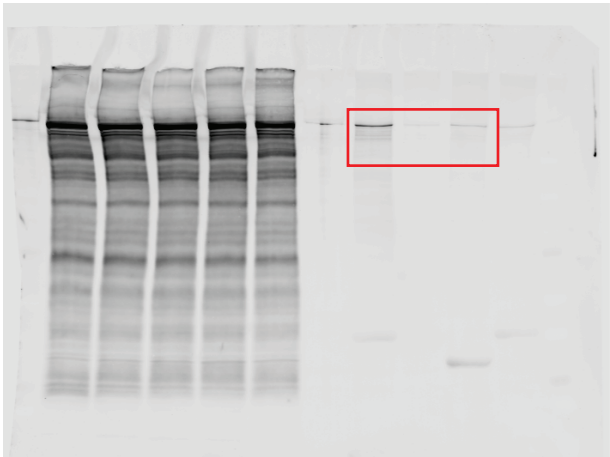

IB: FLAG

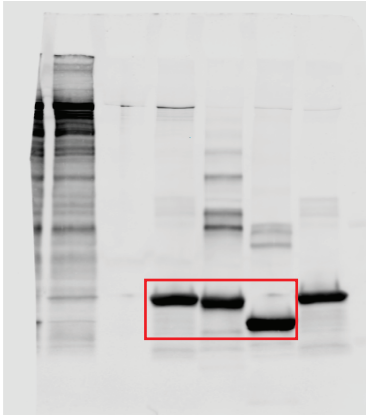

IB: GST

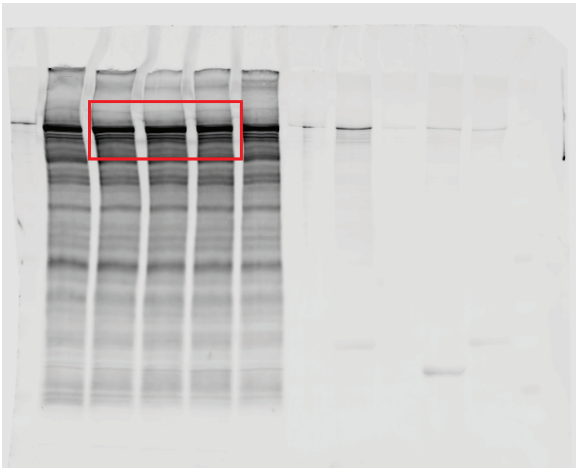

IB: FLAG

Supplement: Supplementary file 6 — Source Data [file 41467_2021_26061_MOESM6_ESM.zip › Source data_2021.08.26/Blots/Figure 1/Figure 1G.pdf]

# Figure 1

## Panel H

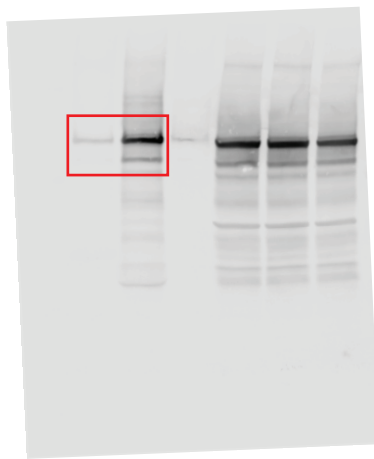

IB: FLAG

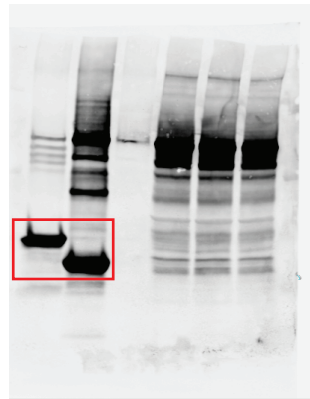

IB: GST

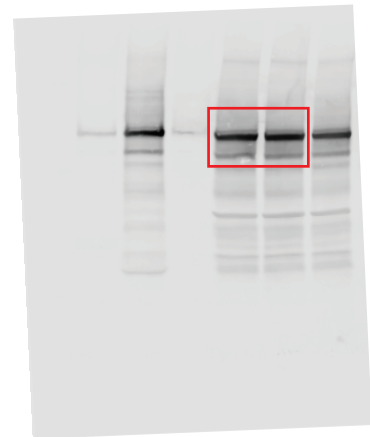

IB: FLAG

Supplement: Supplementary file 6 — Source Data [file 41467_2021_26061_MOESM6_ESM.zip › Source data_2021.08.26/Blots/Figure 1/Figure 1H.pdf]

# Figure 1

## Panel I

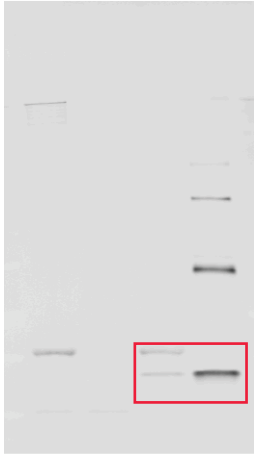

IB: HA

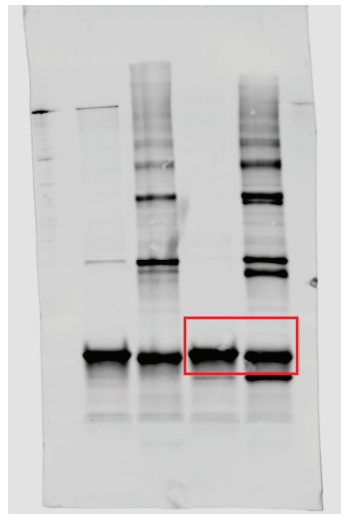

IB: GST

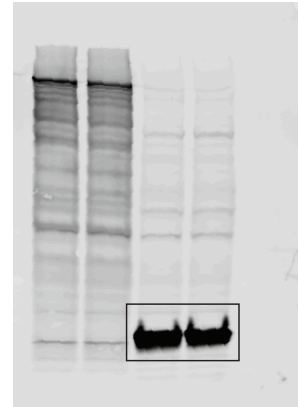

IB: HA

Supplement: Supplementary file 6 — Source Data [file 41467_2021_26061_MOESM6_ESM.zip › Source data_2021.08.26/Blots/Figure 1/Figure 1I.pdf]

Figure 2

Panel A

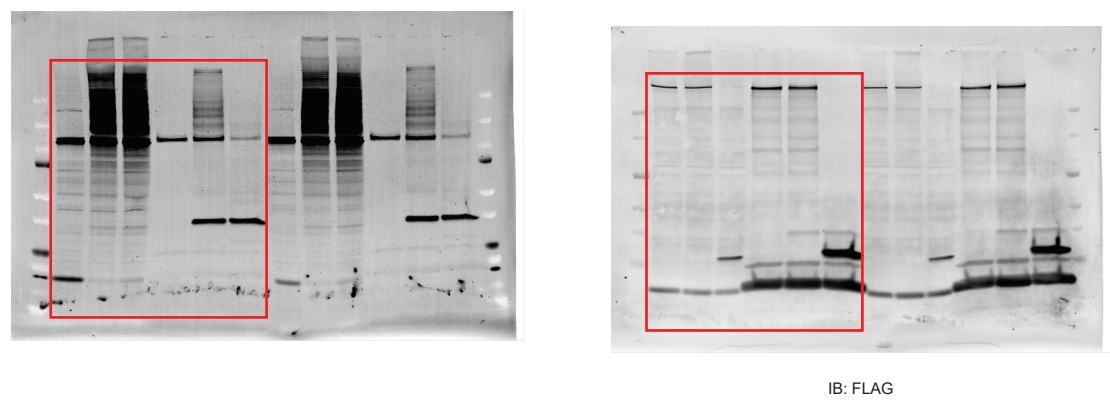

Supplement: Supplementary file 6 — Source Data [file 41467_2021_26061_MOESM6_ESM.zip › Source data_2021.08.26/Blots/Figure 2/Figure 2A.pdf]

# Figure 2

## Panel B

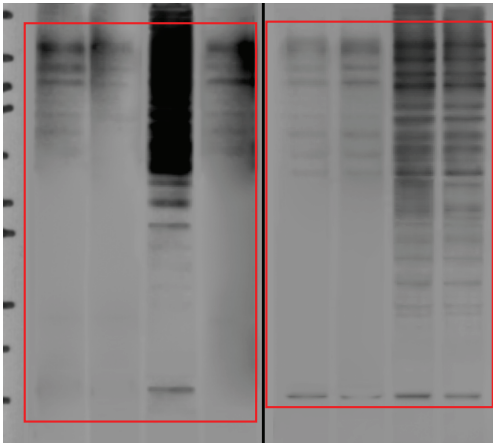

IB: ISG15

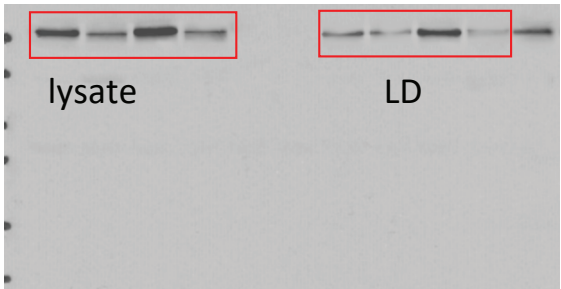

IB: RNF213

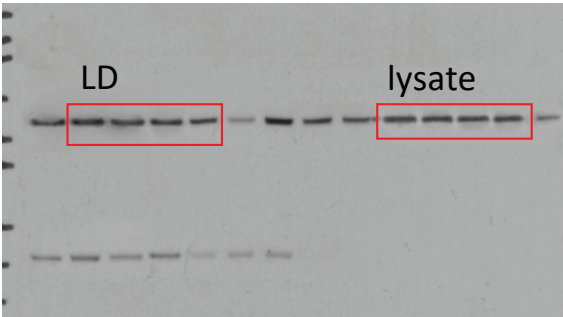

IB: Perilipin 1

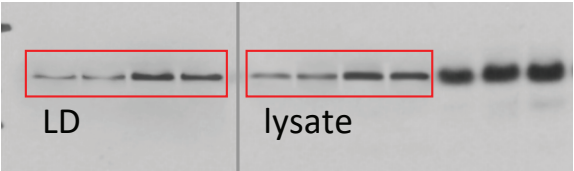

IB: Perilipin 2

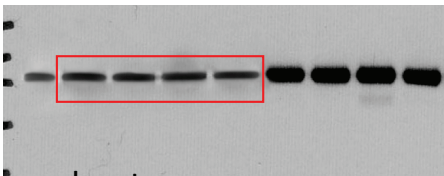

IB: GAPDH

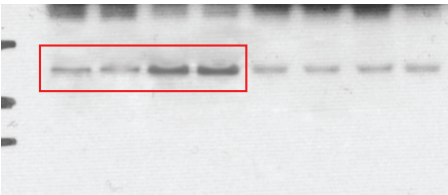

IB: ATGL

Supplement: Supplementary file 6 — Source Data [file 41467_2021_26061_MOESM6_ESM.zip › Source data_2021.08.26/Blots/Figure 2/Figure 2C.pdf]

# Figure 3

## Panel A (top)

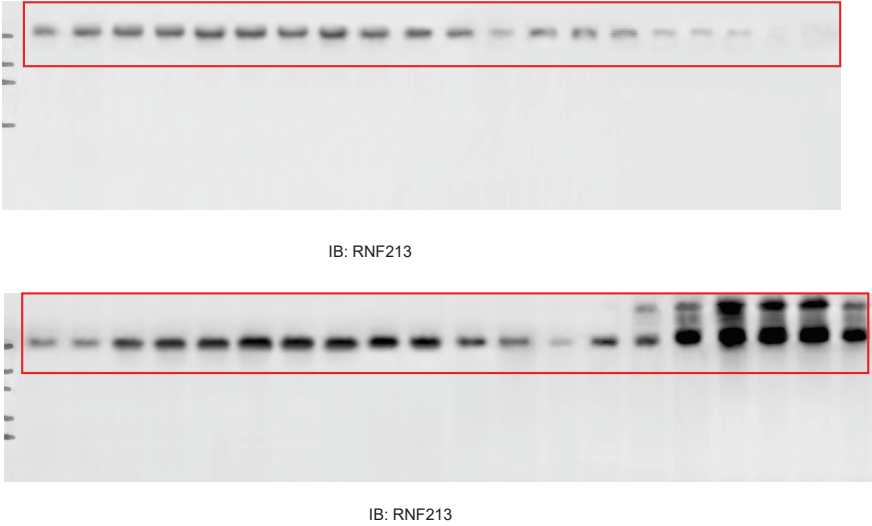

## Panel A (middle)

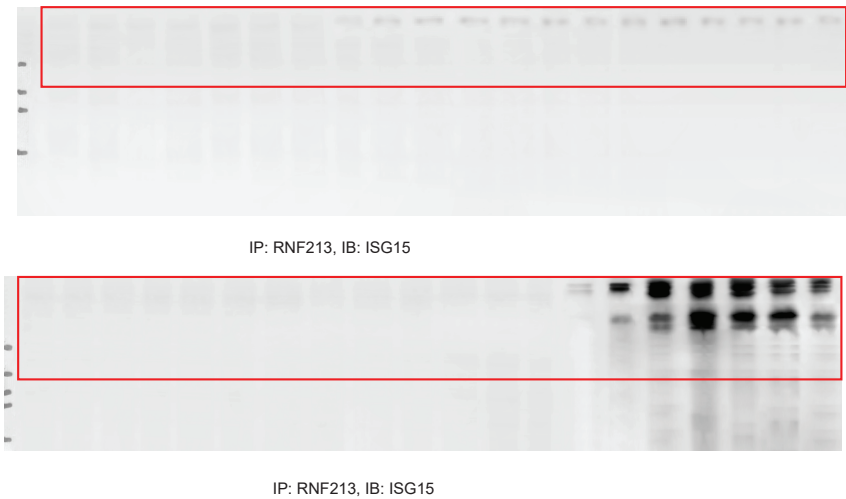

## Panel A (bottom)

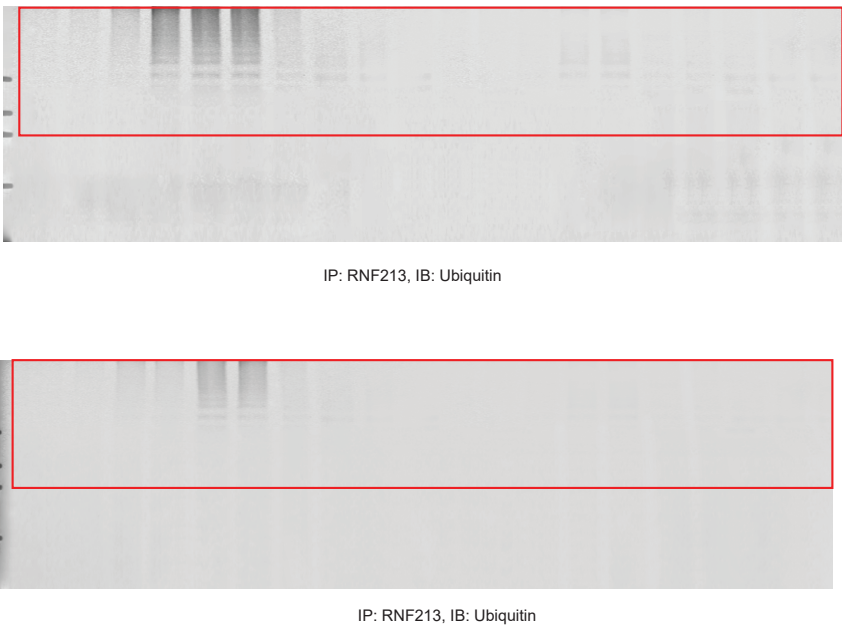

Supplement: Supplementary file 6 — Source Data [file 41467_2021_26061_MOESM6_ESM.zip › Source data_2021.08.26/Blots/Figure 3/Figure 3A.pdf]

# Figure 3

## Panel B

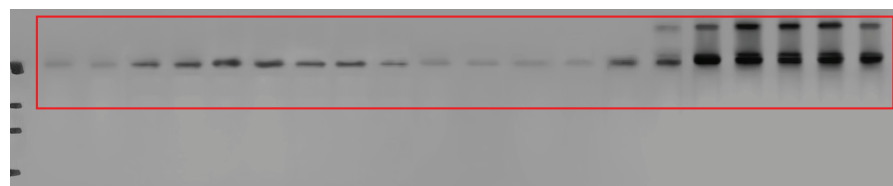

IB: RNF213 (NT)

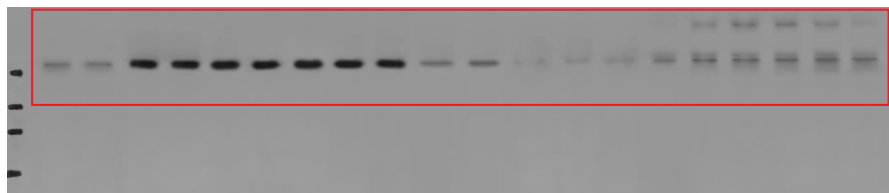

IB: RNF213 (siUbe1L)

Supplement: Supplementary file 6 — Source Data [file 41467_2021_26061_MOESM6_ESM.zip › Source data_2021.08.26/Blots/Figure 3/Figure 3B.pdf]

# Figure 3

## Panel C

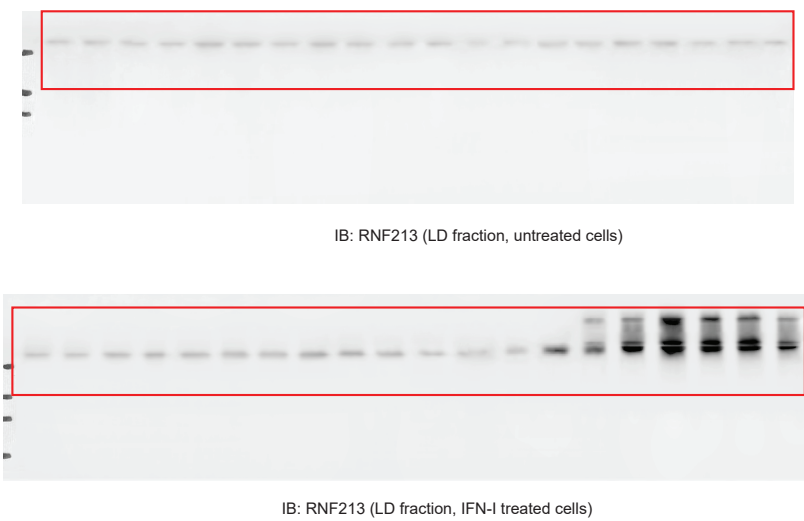

Supplement: Supplementary file 6 — Source Data [file 41467_2021_26061_MOESM6_ESM.zip › Source data_2021.08.26/Blots/Figure 3/Figure 3C.pdf]

Figure 4  
Panel C

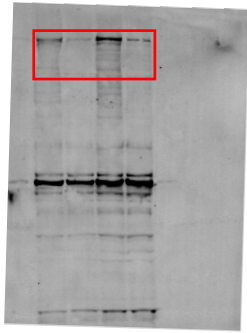

IB: RNF213

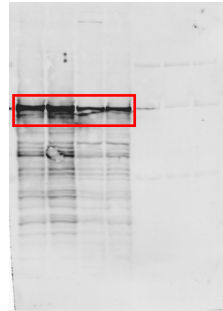

IB: HSV-VP5

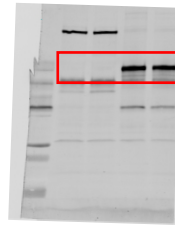

IB: MXA

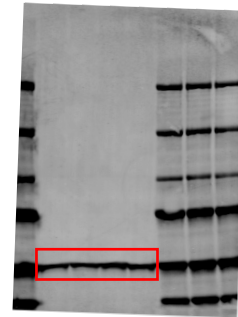

IB: Tubulin

Supplement: Supplementary file 6 — Source Data [file 41467_2021_26061_MOESM6_ESM.zip › Source data_2021.08.26/Blots/Figure 4/Figure 4C.pdf]

Figure 4  
Panel E

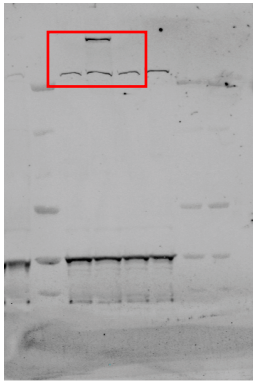

IB: FLAG

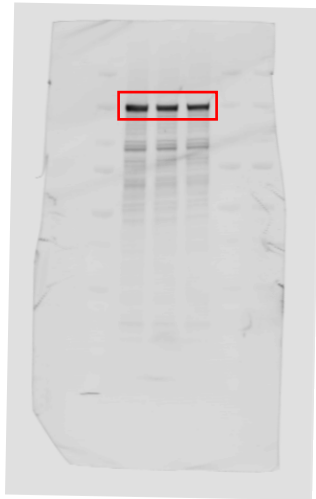

IB: HSV-VP5

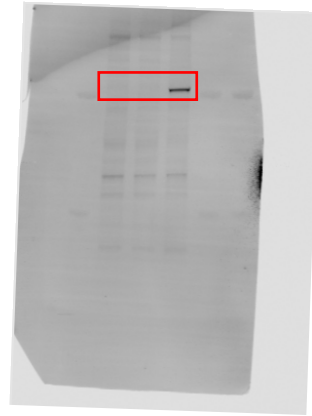

IB: MXB

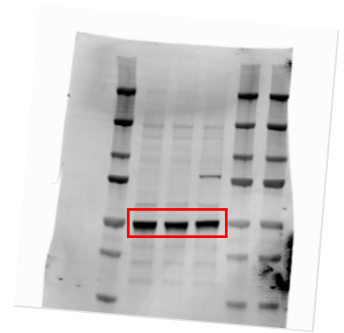

IB: Tubulin

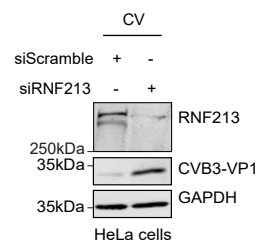

Supplement: Supplementary file 6 — Source Data [file 41467_2021_26061_MOESM6_ESM.zip › Source data_2021.08.26/Blots/Figure 4/Figure 4E.pdf]

# Figure 6

## Panel B

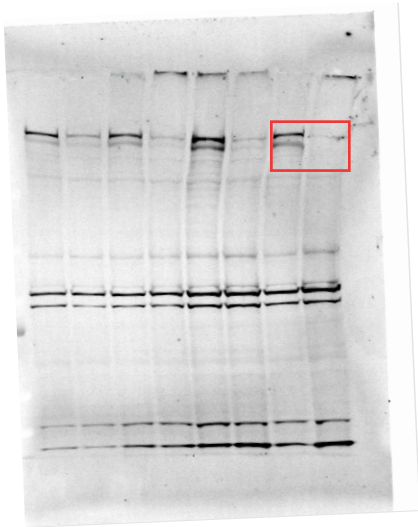

IB: RNF213

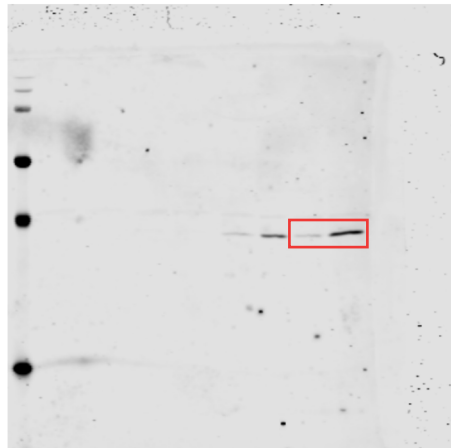

IB: CVB3-VP1

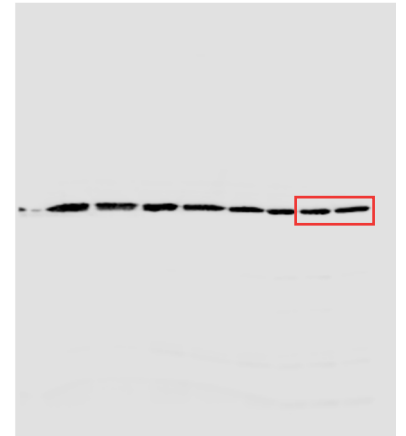

IB: GAPDH

Supplement: Supplementary file 6 — Source Data [file 41467_2021_26061_MOESM6_ESM.zip › Source data_2021.08.26/Blots/Figure 6/Figure 6B.pdf]

Figure 7

Panel A

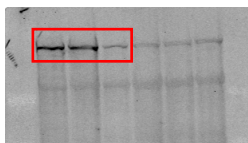

IB: RNF213

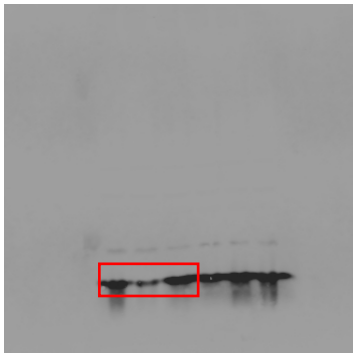

IB: ISG15

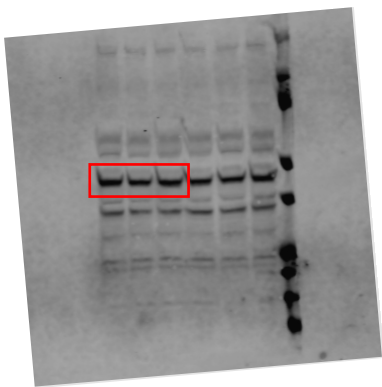

IB: Tubulin

Supplement: Supplementary file 6 — Source Data [file 41467_2021_26061_MOESM6_ESM.zip › Source data_2021.08.26/Blots/Figure 7/Figure 7A.pdf]

Figure 7

Panel C (left)

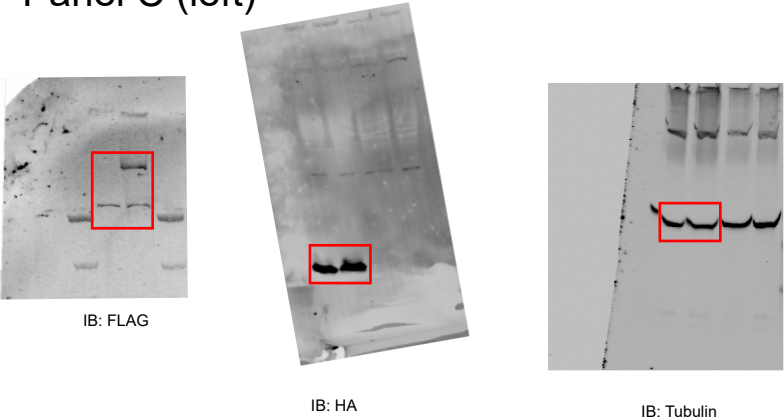

Panel C (right)

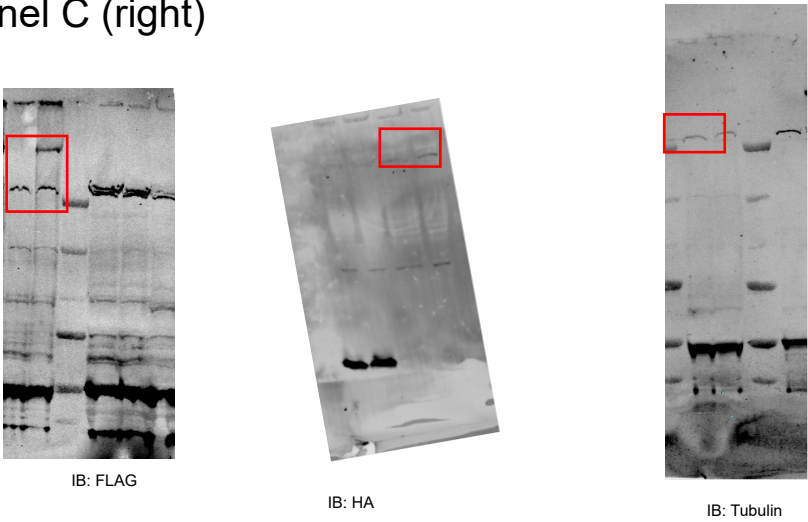

Supplement: Supplementary file 6 — Source Data [file 41467_2021_26061_MOESM6_ESM.zip › Source data_2021.08.26/Blots/Figure 7/Figure 7C.pdf]

Figure 7

Panel D (left)

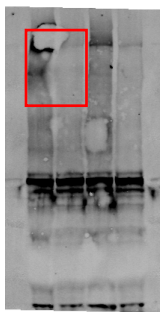

IB: FLAG

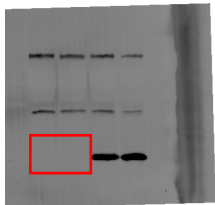

IB: ISG15

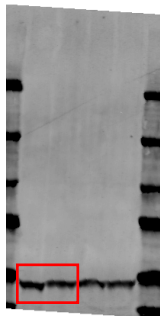

IB: Tubulin

Panel D (right)

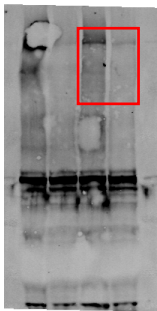

IB: RNF213

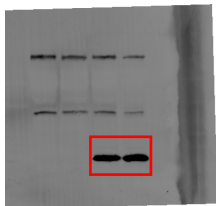

IB: HA

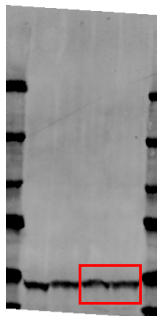

IB: Tubulin

Supplement: Supplementary file 6 — Source Data [file 41467_2021_26061_MOESM6_ESM.zip › Source data_2021.08.26/Blots/Figure 7/Figure 7D.pdf]

Supplementary figure 4 (related to figure 2)

Panel A

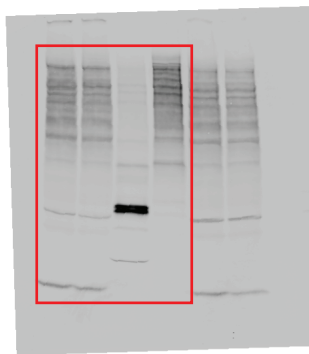

IB: HA

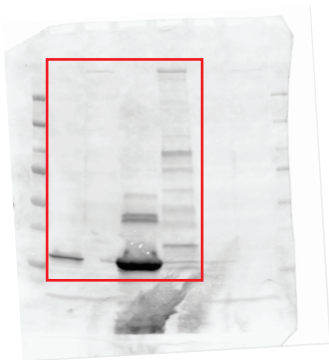

IB: eGFP

Supplement: Supplementary file 6 — Source Data [file 41467_2021_26061_MOESM6_ESM.zip › Source data_2021.08.26/Blots/Supplementary Figure 4/Supplementary figure 4 - Panel A.pdf]

Supplementary figure 4 (related to figure 2)

Panel D (left)

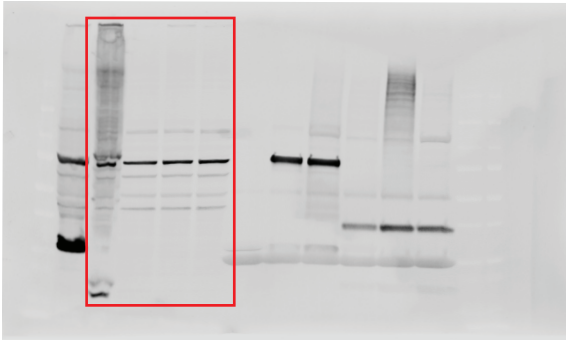

IB: HA

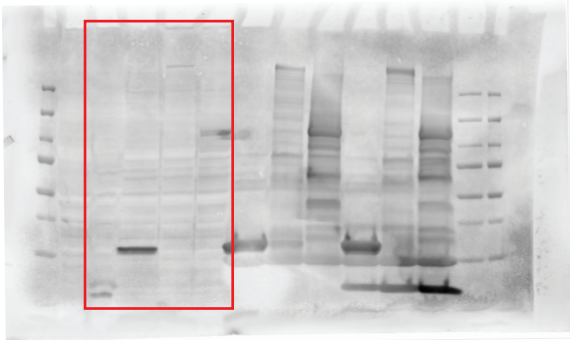

IB: FLAG

Panel D (right)

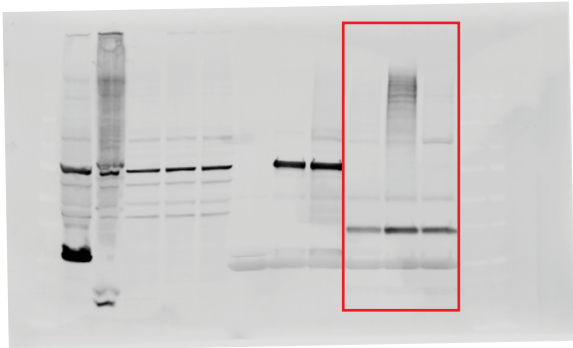

IB: HA

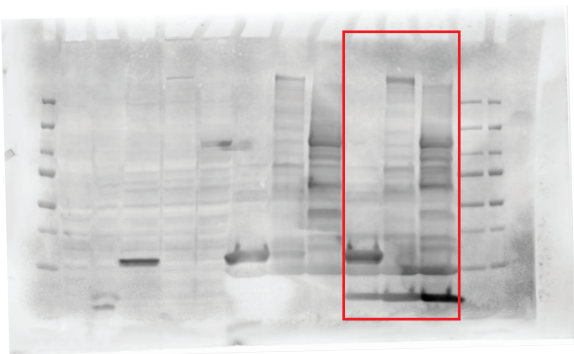

IB: FLAG

Supplement: Supplementary file 6 — Source Data [file 41467_2021_26061_MOESM6_ESM.zip › Source data_2021.08.26/Blots/Supplementary Figure 4/Supplementary figure 4 - Panel D.pdf]

Supplementary figure 4 (related to figure 2)

Panel E (left)

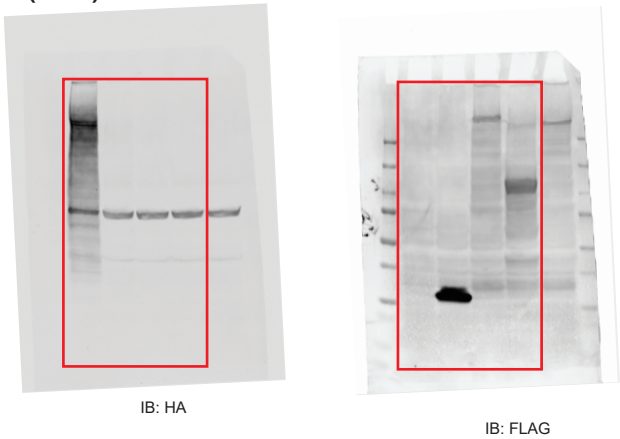

Panel E (right)

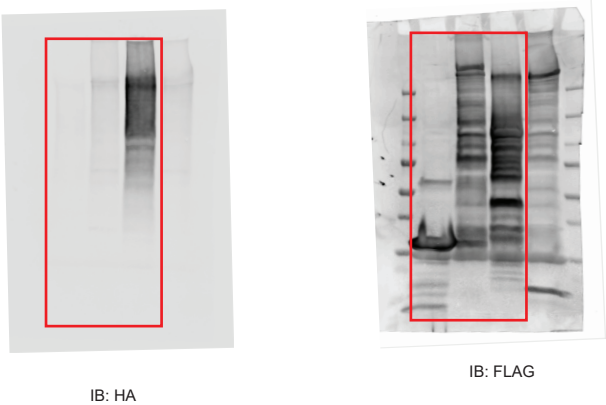

Supplement: Supplementary file 6 — Source Data [file 41467_2021_26061_MOESM6_ESM.zip › Source data_2021.08.26/Blots/Supplementary Figure 4/Supplementary figure 4 - Panel E.pdf]

Supplementary figure 4 (related to figure 2)

Panel F

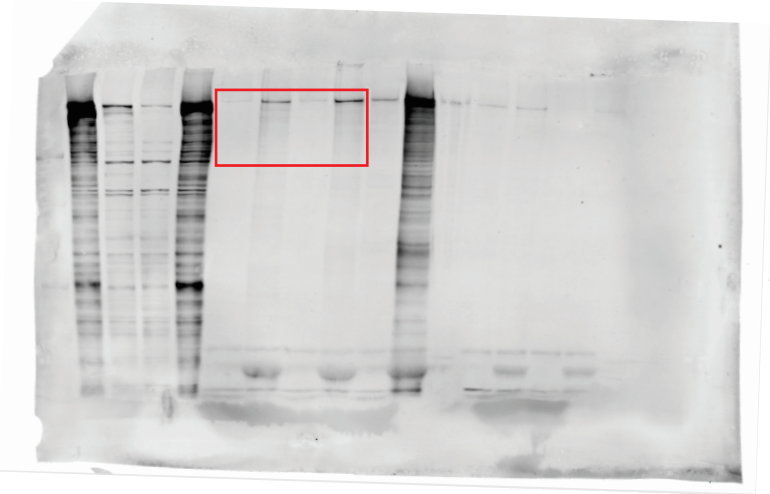

IB: FLAG

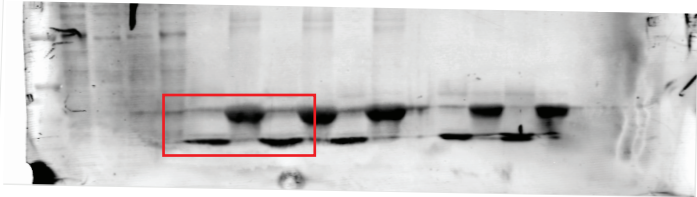

IB: GST

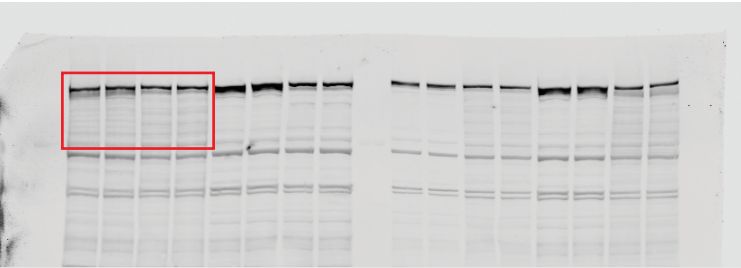

IB: FLAG

Supplement: Supplementary file 6 — Source Data [file 41467_2021_26061_MOESM6_ESM.zip › Source data_2021.08.26/Blots/Supplementary Figure 4/Supplementary figure 4 - Panel F.pdf]

Supplementary figure 5 (related to figure 2)

Panel A

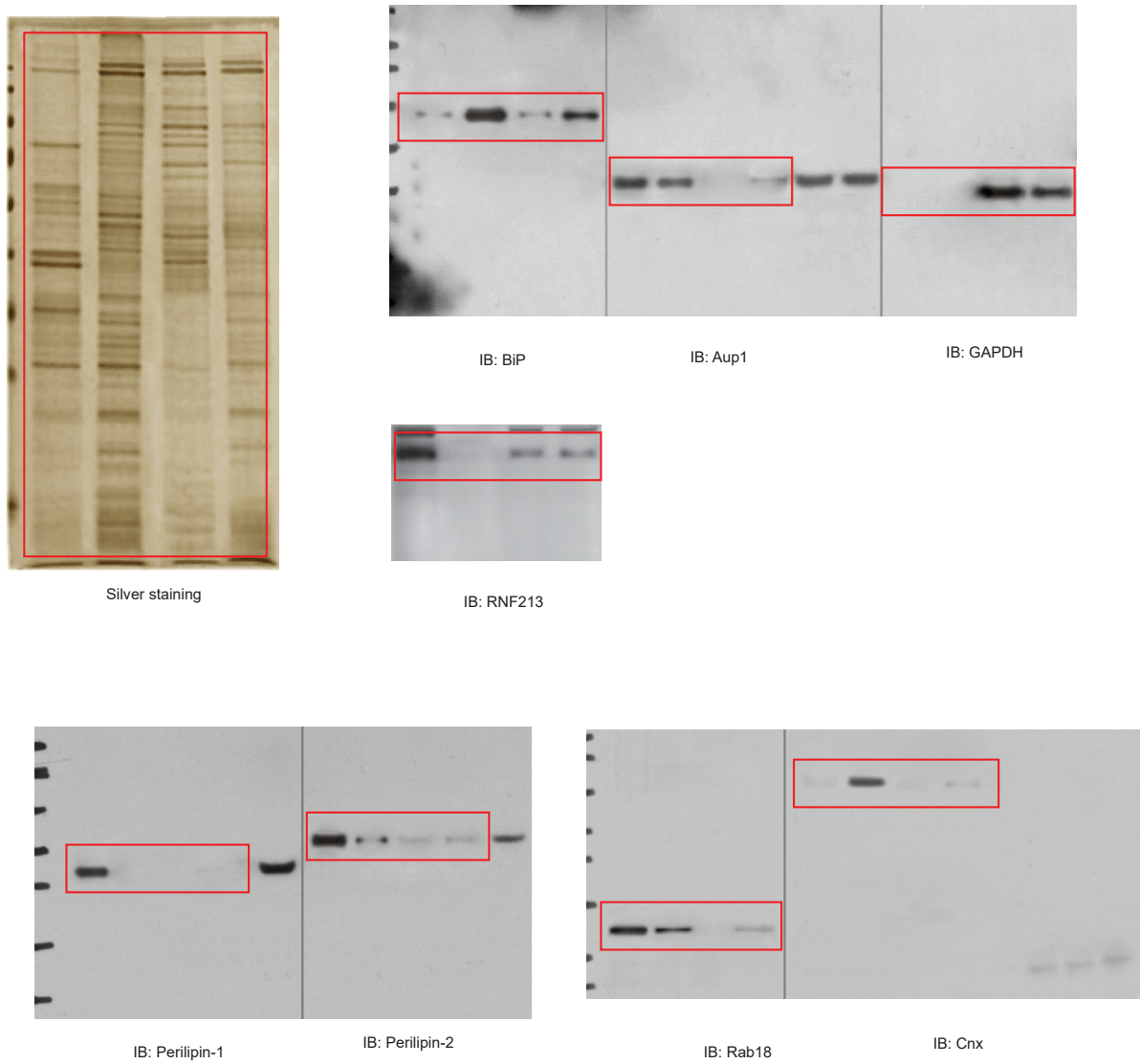

Supplement: Supplementary file 6 — Source Data [file 41467_2021_26061_MOESM6_ESM.zip › Source data_2021.08.26/Blots/Supplementary Figure 5/Supplementary figure 5 - Panel A.pdf]

Supplementary figure 6 (related to figure 3)

Panel A (left)

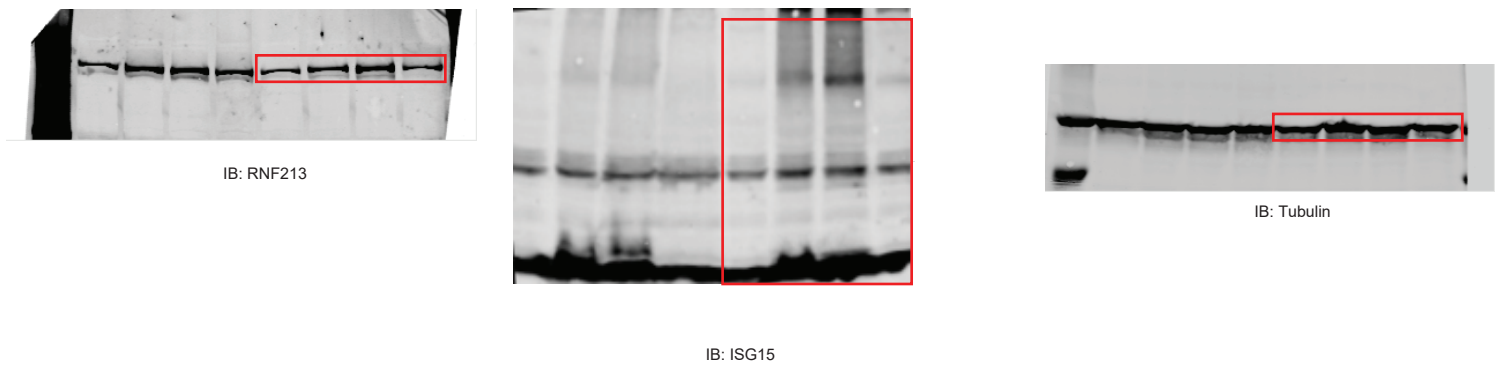

Panel A (middle)

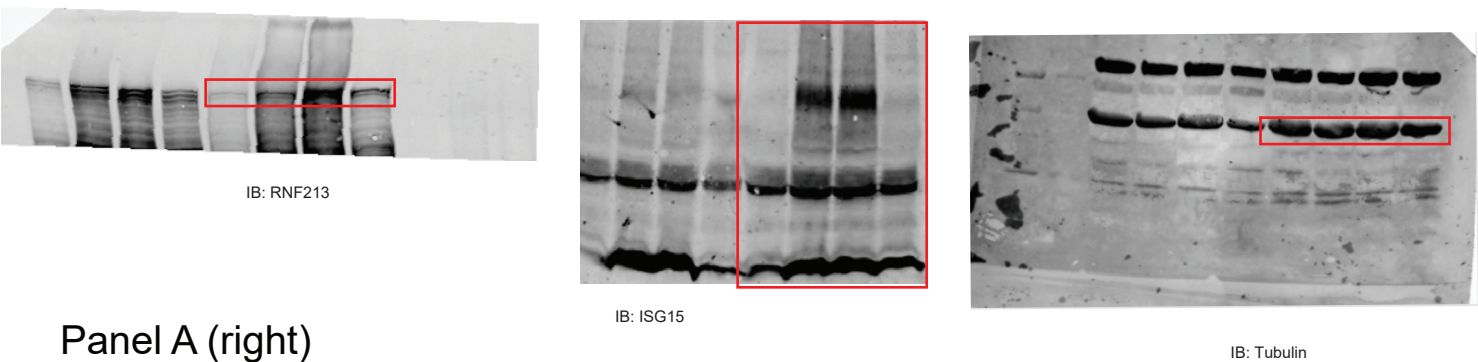

Panel A (right)

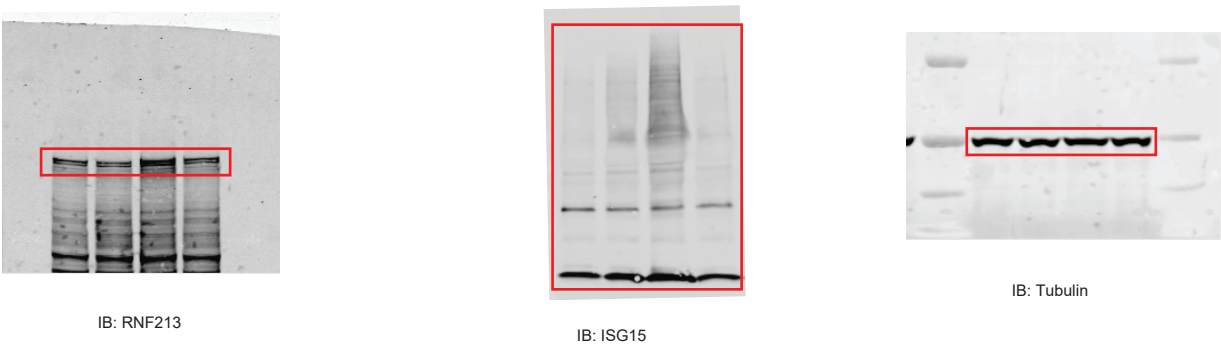

Supplement: Supplementary file 6 — Source Data [file 41467_2021_26061_MOESM6_ESM.zip › Source data_2021.08.26/Blots/Supplementary Figure 6/Supplementary Figure 6 - panel A.pdf]

Supplementary figure 6 (related to figure 3)

Panel B

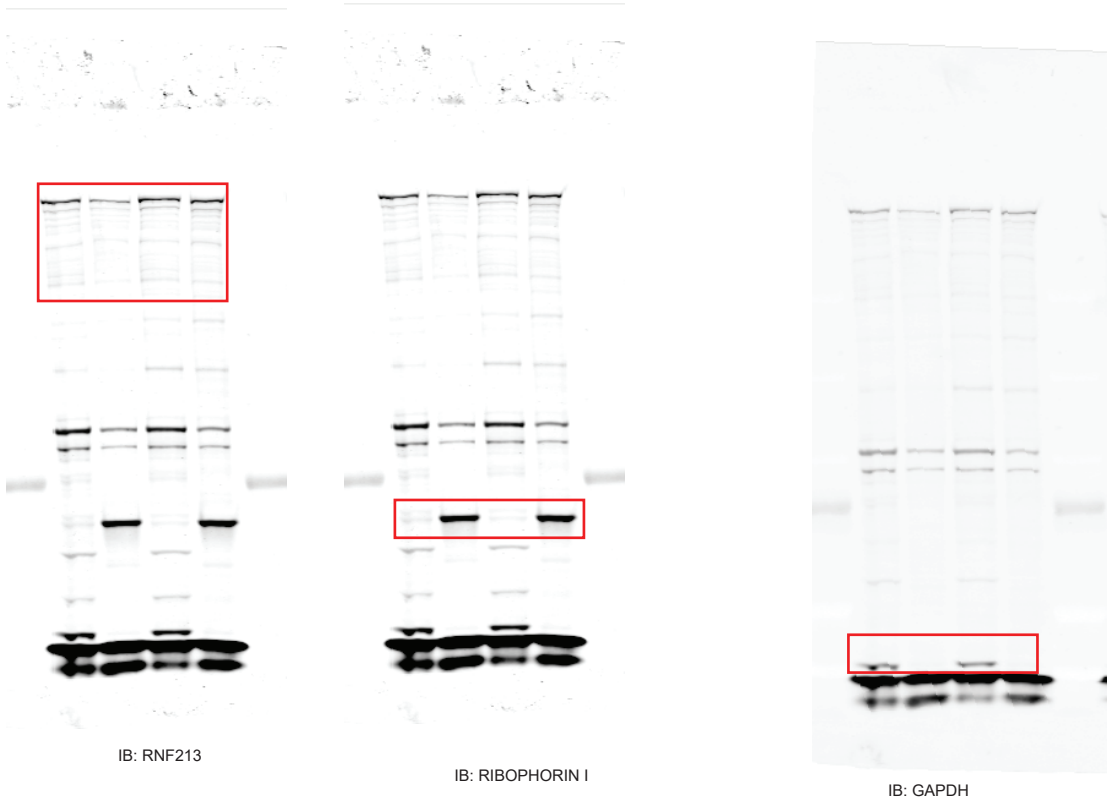

Supplement: Supplementary file 6 — Source Data [file 41467_2021_26061_MOESM6_ESM.zip › Source data_2021.08.26/Blots/Supplementary Figure 6/Supplementary Figure 6 - panel B.pdf]

## Supplementary figure 6 (related to figure 3)

### Panel C

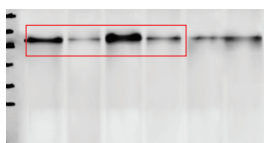

IB: UBE1L

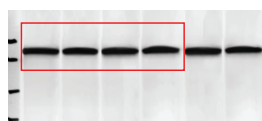

IB: Actin

Supplement: Supplementary file 6 — Source Data [file 41467_2021_26061_MOESM6_ESM.zip › Source data_2021.08.26/Blots/Supplementary Figure 6/Supplementary Figure 6 - panel C..pdf]

## Supplementary Figure 7

### Panel A

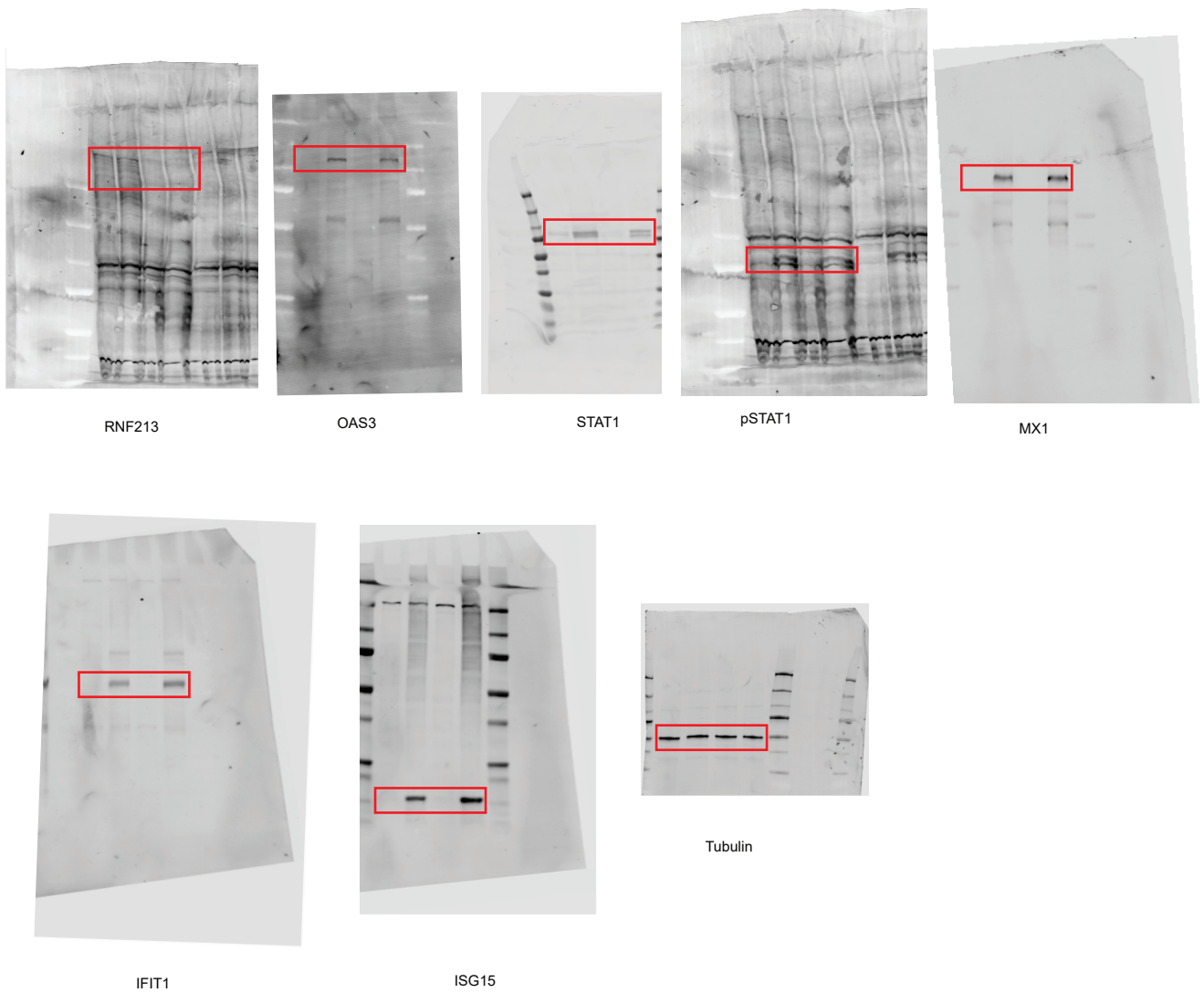

Supplement: Supplementary file 6 — Source Data [file 41467_2021_26061_MOESM6_ESM.zip › Source data_2021.08.26/Blots/Supplementary Figure 7/Supplementary Figure 7 - Panel A.pdf]

## Supplementary Figure 7

### Panel B

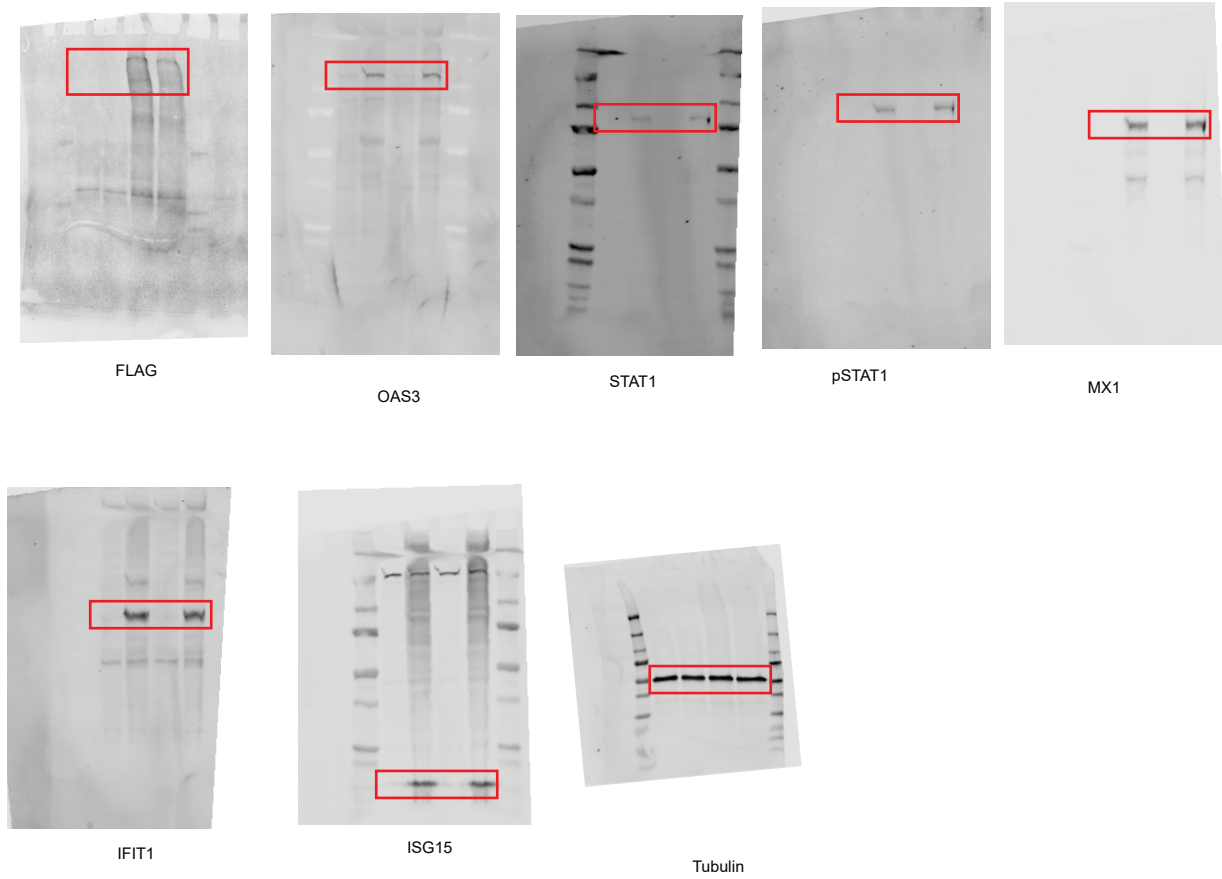

Supplement: Supplementary file 6 — Source Data [file 41467_2021_26061_MOESM6_ESM.zip › Source data_2021.08.26/Blots/Supplementary Figure 7/Supplementary Figure 7 - Panel B.pdf]

## Supplementary Figure 7

### Panel E

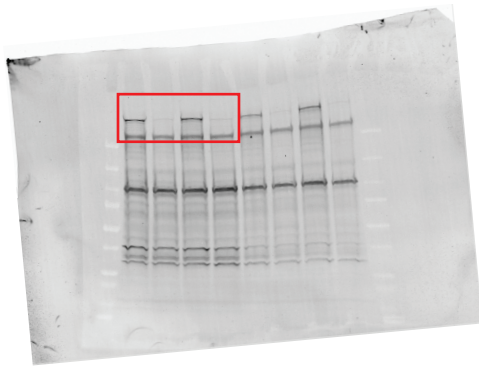

RNF213

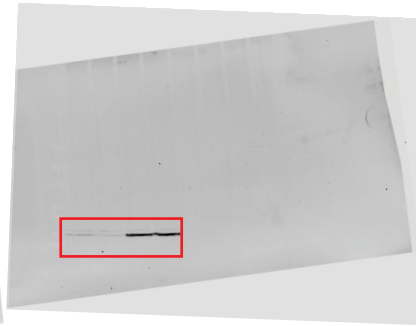

ISG15

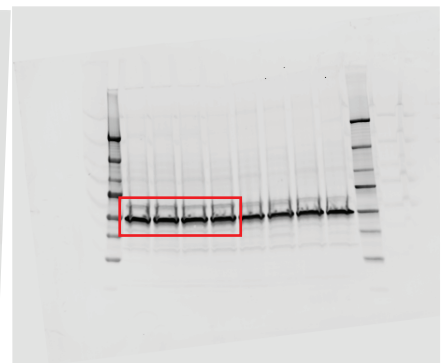

Tubulin

Supplement: Supplementary file 6 — Source Data [file 41467_2021_26061_MOESM6_ESM.zip › Source data_2021.08.26/Blots/Supplementary Figure 7/Supplementary Figure 7 - Panel E.pdf]

## Supplementary Figure 7

### Panel H

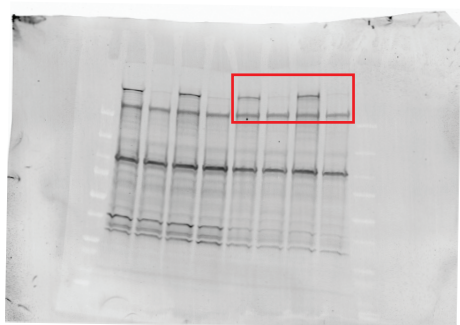

RNF213

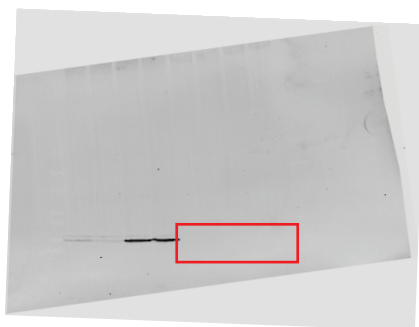

ISG15

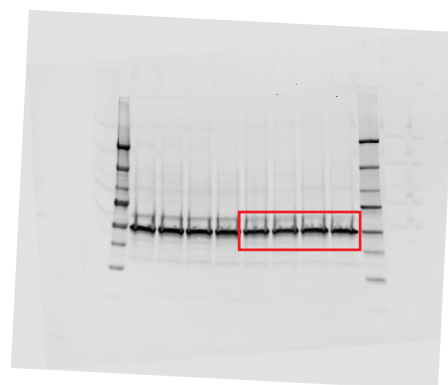

Tubulin

Supplement: Supplementary file 6 — Source Data [file 41467_2021_26061_MOESM6_ESM.zip › Source data_2021.08.26/Blots/Supplementary Figure 7/Supplementary Figure 7 - Panel H.pdf]

## Supplementary Figure 8

### Panel A

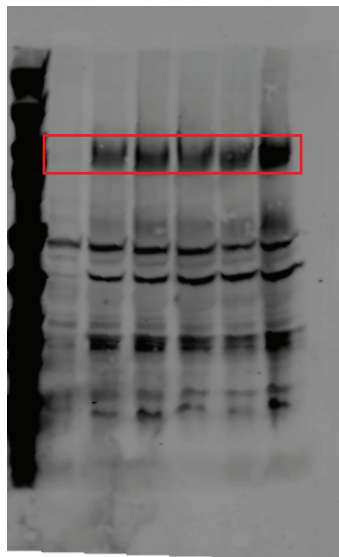

IB: RSV-G

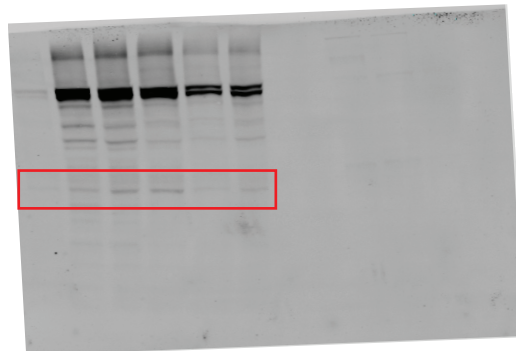

IB: pSTAT1

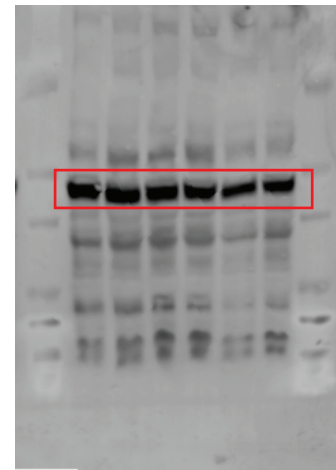

IB: Tubulin

Supplement: Supplementary file 6 — Source Data [file 41467_2021_26061_MOESM6_ESM.zip › Source data_2021.08.26/Blots/Supplementary Figure 8/Supplementary Figure 8 - Panel A.pdf]

## Supplementary Figure 8

### Panel B

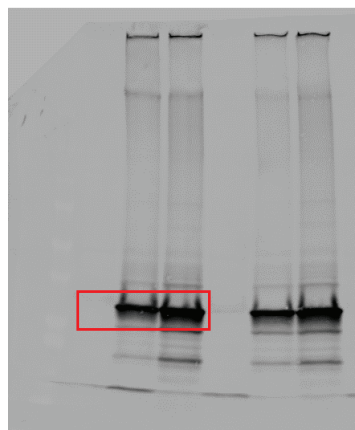

IB: CVB3-VP1

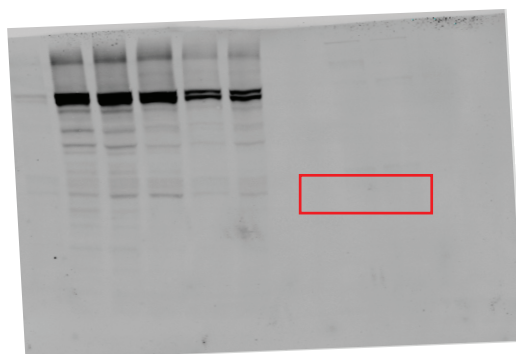

IB: pSTAT1

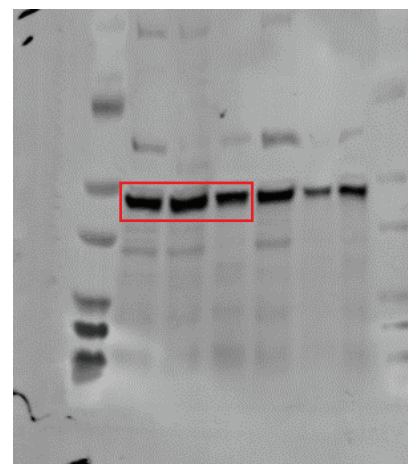

IB: Tubulin

Supplement: Supplementary file 6 — Source Data [file 41467_2021_26061_MOESM6_ESM.zip › Source data_2021.08.26/Blots/Supplementary Figure 8/Supplementary Figure 8 - Panel B.pdf]

## Supplementary Figure 8

### Panel D

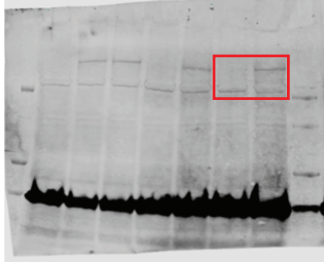

IB: FLAG

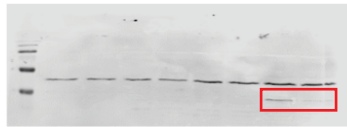

IB: CV-VP1

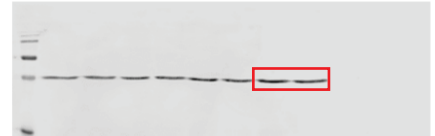

IB: GAPDH

Supplement: Supplementary file 6 — Source Data [file 41467_2021_26061_MOESM6_ESM.zip › Source data_2021.08.26/Blots/Supplementary Figure 8/Supplementary Figure 8 - Panel D.pdf]

## Supplementary Figure 9

### Panel A

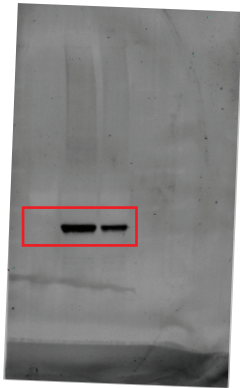

IB: EF-TU

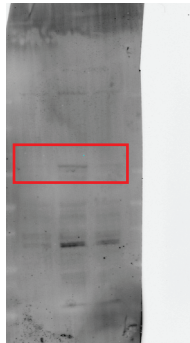

IB: pSTAT1

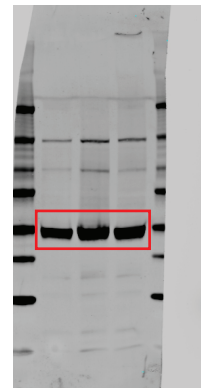

IB: Tubulin

Supplement: Supplementary file 6 — Source Data [file 41467_2021_26061_MOESM6_ESM.zip › Source data_2021.08.26/Blots/Supplementary Figure 9/Supplementary Figure 9 - Panel A.pdf]
